# Discovery of Potential New Gene Variants and Inflammatory Cytokine Associations with Fibromyalgia Syndrome by Whole Exome Sequencing

**DOI:** 10.1371/journal.pone.0065033

**Published:** 2013-06-10

**Authors:** Jinong Feng, Zhifang Zhang, Xiwei Wu, Allen Mao, Frances Chang, Xutao Deng, Harry Gao, Ching Ouyang, Kenneth J. Dery, Keith Le, Jeffrey Longmate, Claudia Marek, R. Paul St. Amand, Theodore G. Krontiris, John E. Shively

**Affiliations:** 1 Department of Immunology, Beckman Research Institute, City of Hope, Duarte, California, United States of America; 2 Department of Molecular Medicine, Beckman Research Institute, City of Hope, Duarte, California, United States of America; 3 Department of Biostatistics, Beckman Research Institute, City of Hope, Duarte, California, United States of America; 4 DNA Sequencing Core, Beckman Research Institute, City of Hope, Duarte, California, United States of America; 5 R.P. St. Amand MD Inc, Marina Del Rey, California, United States of America; Yale School of Public Health, United States of America

## Abstract

Fibromyalgia syndrome (FMS) is a chronic musculoskeletal pain disorder affecting 2% to 5% of the general population. Both genetic and environmental factors may be involved. To ascertain in an unbiased manner which genes play a role in the disorder, we performed complete exome sequencing on a subset of FMS patients. Out of 150 nuclear families (trios) DNA from 19 probands was subjected to complete exome sequencing. Since >80,000 SNPs were found per proband, the data were further filtered, including analysis of those with stop codons, a rare frequency (<2.5%) in the 1000 Genomes database, and presence in at least 2/19 probands sequenced. Two nonsense mutations, W32X in C11orf40 and Q100X in *ZNF77* among 150 FMS trios had a significantly elevated frequency of transmission to affected probands (p = 0.026 and p = 0.032, respectively) and were present in a subset of 13% and 11% of FMS patients, respectively. Among 9 patients bearing more than one of the variants we have described, 4 had onset of symptoms between the ages of 10 and 18. The subset with the C11orf40 mutation had elevated plasma levels of the inflammatory cytokines, MCP-1 and IP-10, compared with unaffected controls or FMS patients with the wild-type allele. Similarly, patients with the *ZNF77* mutation have elevated levels of the inflammatory cytokine, IL-12, compared with controls or patients with the wild type allele. Our results strongly implicate an inflammatory basis for FMS, as well as specific cytokine dysregulation, in at least 35% of our FMS cohort.

## Introduction

FMS is characterized by chronic, widespread pain in the muscles and joints. In addition, patients with FMS often report fatigue, non-restorative sleep, cognitive dysfunction, stiffness, and mood disturbance [1]. The prevalence of FMS in the general population is estimated at 2% to 5%, of which 85% are females [2]. Although the exact causes of FMS are not fully understood, evidence suggests that both genetic and environmental factors are involved. Studies in search of the genetic predisposition to FMS have been conducted based on the strong evidence of a familial aggregation in FMS [3]–[6]. Though several lines of evidence suggest a role for polymorphisms of genes in the serotoninergic, catecholaminergic and dopaminergic systems in the pathogenesis of FMS, these polymorphisms are not specific for FMS and are similarly associated with additional co-morbid conditions [6]–[8]. The lack of evidence for a Mendelian, monogenic mode of transmission suggests that multiple genes might influence the onset of FMS [9].

Several groups, including our own, have taken a candidate gene approach. Since FMS is associated with widespread pain, one study analyzed over 350 genes associated with pain and found an association with 4 genes [10]. In that study, the underlying assumption was that FMS is a pain disorder. However, it is possible that the widespread pain observed in FMS is caused by an underlying factor such as chronic inflammation and that defects in pain-related genes alone are not the cause of the syndrome.

In a search for possible candidate genes for FMS with an inflammatory basis, we became interested in the *MEFV* gene, in which a number of mutations cause Familial Mediterranean Fever (FMF). FMS and FMF share some overlapping symptoms, including unexplained abdominal pain in FMF [11] and high prevalence of irritable bowel syndrome in FMS [12], [13], both of which suggest an inflammatory condition. To explore the possibility that variants of the *MEFV* gene may predispose to FMS, we sequenced exons and splice junctions as regions of likely functional significance in *MEFV* in 100 FMS probands and their parents. Our data provided evidence that rare missense variants of the *MEFV* gene are collectively associated with risk of FMS and are present in a subset of about 15% of FMS patients. In addition, the subset of FMS patients with these MEFV variants had elevated plasma levels of IL-1β, the main cytokine associated with the *MEFV* (or pyrin) gene [14]. These findings supported our hypothesis regarding a genetic link to an inflammatory basis for this syndrome.

In the present study, we have broadened our analysis by sequencing the entire exomes of 19 FMS probands out of a larger cohort of 150 unrelated FMS patients and subsequent directed mutation analysis in the remaining 131 FMS patients. We identified two promising genes associated with FMS, one of which correlates with high plasma levels of MCP-1 and IP-10, and the other with high levels of IL-12, further supporting a probable inflammatory basis of the syndrome.

## Materials and Methods

### Patient Characteristics

This study including the written informed consent form was approved by the Institutional Review Board of City of Hope National Medical Center (IRB 04186). A total of 150 patients with fibromyalgia and their parents were recruited into the study. Written informed consent was obtained from all participants. Patients were diagnosed by including American College of Rheumatology criteria [15]. Musculoskeletal pains had existed for over three months and were accompanied by complaints from one or more other systems: central nervous (fatigue, depression, cognitive difficulty); gastrointestinal (irritable bowel); genitourinary tract (dysuria, frequency, interstitial cystitis). Using digital pressure on eighteen predetermined sites located in each quadrant of the body, at least eleven tender points were elicited. Patients with the autoimmune diseases, rheumatoid arthritis and systemic lupus erythematosus, were excluded from the study; other clinical characteristics of the patient population have been previously described [16]. **Table 1** lists the characteristics of the 150 probands. Unrelated, age-matched, control subjects, as previously described [16], were used for comparison of cytokine/chemokine levels; but the genetic analysis used the transmission disequilibrium test, which avoids the need for unaffected control subjects.

**Table 1:** Characteristics of FMS probands (N = 150).

Demographic characteristics	
Sex-no.(%)	
Female	119 (79%)
Male	31 (21%)
Age– yr	
Mean (±SD)	33±15
Range	10–68
Time since diagnosis-yr	
Mean (±SD)	7.2±4.6
Range	2–33
Family history-no.(%)	
Positive	107 (71%)
Negative	43 (29%)
Ethnicity-no.(%)	
European/Caucasian	135 (90%)
Asian	11 (7%)
Hispanic	4 (3%)
**Clinical characteristics** 1	
Pain	148 (99%)
Fatigue	139 (93%)
Headache	129 (86%)
Irritability	130 (87%)
Insomnia	106 (71%)

1 Patientg001s meet principal symptoms for ACR diagnostic criteria^21^.

### Exome Sequencing

Genomic DNA from 19 FMS probands was extracted from peripheral blood lymphocytes using standard protocols and sonicated (Bioruptor, Diagenode Inc., Denville, NJ) for 13 cycles. Sonicated DNA (3 µg) was purified, and Illumina adapters were added to make a library for paired-end sequencing (Illumina Inc., San Diego, CA). Fragments with approximately 200–250 bp insert DNA were selected and amplified. The library (750 ng) was hybridized to biotinylated cRNA oligonucleotide baits from the SureSelect Human All Exon kit (Agilent Technologies Inc., Santa Clara, CA), purified by streptavidin-bound magnetic beads, and amplified for 12 cycles. After purification, the library was paired-end (80×80 bp) sequenced using an Illumina Genome Analyzer IIx or Hiseq 2000 (Illumina Inc., San Diego, CA). The exome probes covered 50 Mb of the human genome, corresponding to the exons and flanking intronic regions of all genes in the National Center for Biotechnology Information Consensus CDS database, 700 miRNAs from the Sanger v13 database, and 300 noncoding RNAs from Ensembl GRCh37.56. The data set has been deposited with the NIH short read archive with accession no. SRA072282.

### PCR and Sequencing

DNA was isolated from peripheral leukocytes or saliva, using the QIAamp DNA Blood Mini Kit (Qiagen) and Oragene DNA Self Collection Kits (DNA Genotek) according to the manufacturers' instructions. The coding exons with known SNPs from deep sequencing were amplified by PCR in a total volume of 20 µl with 10 mM Tris-HCl, pH 8.3, 50 mM KCl, 1.5 mM MgCl_2_, 200 µM of each deoxyribonucleoside triphosphate, and 0.2 µM of primers. 1 U of Ampli-Taq Gold (Roche) and 20 ng of genomic DNA were added. PCR reactions were performed on the GeneAmp PCR System 9700 (Applied Biosystems) with denaturation at 94°C for 10 min, and then denaturation at 94°C for 15 sec, annealing at 60°C for 30 sec, and elongation at 72°C for 1 min for a total of 35 cycles and a final elongation for 10 min at 72°C. The amplicons were purified by ExoSAP-IT and sequenced with the ABI PRISM 3730 (Applied Biosystems). The sequences of PCR primers are available upon request. Genomic and amino acid sequences for candidate genes were collected from Ensemble.

### DNA Sequence Data Analysis

The paired-end sequences were aligned to the genome (hg19) using Novoalign (http://www.novocraft.com) with default settings. Only reads aligned to a unique genomic location were kept for further analysis. The aligned reads were piled up using Samtools v0.1.12. The subsequent analysis was performed using a custom-developed bioinformatics pipeline implemented using R and Java. The piled-up bases at each genomic position were summarized to identify the potential SNPs using the following criteria: only consider bases with 1) base-calling quality score > = 13; 2) total coverage > = 5; 3) alternative allele coverage > = 2; and 4) alternative allele frequency (AAF) > = 15%. We chose the less stringent criteria because we wanted to maximize the true positive rate, and we further filtered out false positives by aggregating the SNPs in each individual and selecting the ones with highest frequency in the 19 sequenced samples. The potential SNPs identified in each sample were pooled together to generate a master list of potential SNPs in this cohort. For each SNP in the master list, the genotype for each sample was determined by the AAF; e.g., homozygous alternative for samples with AAF > = 85%, homozygous reference for samples with AAF < = 15%, and heterozygous for samples with an AAF in between. The functional consequence of these SNPs was identified using the SeattleSeq annotation site (http://snp.gs.washington.edu/SeattleSeqAnnotation131/). Only SNPs that were missense or nonsense were kept for further analysis. The SNPs were also compared with dbSNP134 (http://www.ncbi.nlm.nih.gov/mailman/htdig/dbsnp-announce/2011q3/000104.html) and the May, 2011 phase I release of the 1000 Genomes project (ftp://ftp-trace.ncbi.nih.gov/1000genomes/ftp/release/20110521/) to differentiate known and novel SNPs. The SNPs annotated to the same refseq gene were aggregated based on the refseq gene symbol, and a frequency table was generated based on the affected gene. The SNPs with the highest frequency were selected for further validation using Sanger sequencing.

### RNA Isolation, RT-PCR, and Real-Time PCR

Samples of normal tissue from the FirstChoice Human Total RNA survey panel were purchased from Applied Biosystems. Monocytes were isolated from human peripheral blood using an EasySep® Human Monocyte Enrichment Kit from StemCell Technologies. U937, THP-1, and HL-60 cell lines were purchased from American Type Culture Collection (ATCC). Total RNA was isolated using the RNeasy isolation kit (Qiagen) and treated with DNase I (Qiagen). Reverse transcription was carried out using 1 µg of total RNA using Oligo(dT)12–18 primer (Invitrogen) and Moloney murine leukemia virus reverse transcriptase (Invitrogen). PCR amplification of C11orf40 and the constitutively expressed housekeeping gene glyceraldehyde phosphate dehydrogenase (GAPDH) transcripts were performed using gene-specific primers (provided upon request). PCR reactions were initiated by heating the samples to 98°C for 30 s followed by 30 cycles of 98°C for 5 s, 60°C for 5 s, and 72°C for 10 s followed by a final extension at 72°C for 1 min. The products were analyzed on 1% agarose gels and visualized by SYBR safeDNAgel stain (Invitrogen). For quantitative real-time PCR, amplification of C11orf40 and GAPDH transcripts was performed using gene-specific primers and SsoFast™ EvaGreen® Supermix (Bio-Rad) at an annealing temperature of 65 °C.

### Transmission Disequilibrium Test (TDT)

The exact version of the TDT [17] was applied to the seven genes emerging from the screening procedure described in the results section, as well as the MEFV gene identified in previous work [14]. Direct sequencing of each of these 7 genes in the 300 parents of our 150 probands identified 13 to 33 parents who were heterozygous for each gene. The number of transmissions of the nonsense or rare variant from heterozygous parents was referred to a binomial distribution with 0.5 probability of transmission to test the null hypothesis of neutrality against the hypothesis of enrichment of variants by sampling of FMS cases. No arbitrary criterion for statistical significance was set (no adjustment for multiple comparisons was made), as significance probabilities were used to select the most promising genes for further evaluation of association with cytokine levels.

### Measurement of Cytokines and Chemokines

The plasma level of cytokines and chemokines of a portion of the cohort described here were previously reported [16]. The levels were compared in unrelated controls and probands and family members with wild type or variant alleles. Statistical analysis was performed by a two-sided, unpaired Student’s t test.

### Human Monocyte Preparation, Transfection and Treatment

Human monocytes were purified from peripheral blood mononuclear cells (PBMCs). Briefly, PBMCs were isolated from citrated blood by centrifugation over Ficoll-Paque Plus (GE healthcare biosciences, Pittsburgh, PA, USA) density gradient. Monocytes were separated from PBMCs using EasySep® Human Monocyte Enrichment Kit (StemCell Technologies Inc, Vancouver, Canada). The purity was >95% as analyzed with anti-CD14-FITC by flow cytometry on a FACSCanton II. Monocytes were transfected with GFP-Vector, GFP-C11orf40, or GFP-W32X using Amaxa® Human Monocyte Nucleofector Kit (Life Technologies, Grand Island, NY). Monocytes (1×10^6^ cells/mL) were suspended in RPMI 1640 medium supplemented with 10% FBS, incubated for 24 hours, then treated with 100 ng/mL LPS (TLR4 agonist), 1 µg/mL Pam3CSK4 (TLR1/2 agonist), or 1 µg/mL CL075 (TLR7/8 agonist) for another 24 hours. Supernatants were collected and analyzed using the Human Cytokine 25-Plex antibody bead kit from Biosource International Inc.

## Results

### Exome Sequencing and SNP Filtering

The mean values for total reads and sequencing yields from exome sequencing of 19 FMS probands were about 6.8×10^7^ reads/per sample and 5.35 Gbp/per sample, respectively, where 3.9×10^7^ reads and 3.13 Gbp/per sample were uniquely aligned to the hg19 genome. The median coverage of targeted exon regions was 31X (72.9% had >10× coverage). The total number of SNPs was about 790,000, of which 66,902 were missense, nonsense, or affecting splicing sites. The average number of SNPs per sample was 82,738. The SNPs had a concordance rate with dbSNP of 96.2%. The summary of the exome sequencing is listed in **Table S1**.

This large number of SNPs precluded analysis of all individual SNPs. Therefore, we developed a filtering strategy with the following steps: (1) Only variants producing missense, nonsense, or splice variants were initially considered (66,902). (2) Of these, only variants found in at least 2 of the 19 FMS patients were taken further (about 18,000 SNPs). (3) Of SNPs satisfying the first two criteria, only SNPs found in the May 2011 release of the 1000 Genomes database (the release available when the above results were obtained) were selected for further analysis (about 12,000 SNPs). (4) Additionally, we restricted this round of analysis to variants with a population frequency in 1000 Genomes of less than or equal to 2.5%, to focus on genes with rare and intermediate AAFs. Under these conditions, 905 SNPs remained (**Table S2)**. (5) Finally, we chose to analyze only those variants likely to produce the most severe effect on phenotype, namely nonsense mutations. Seven variants comprised our candidate gene set after this final filter (**Table 2**).

**Table 2:** TDT analysis of FMS candidate genes^1^.

Rank	Gene	Variants	N	Sequencing Results	FMS Allele Frequency	1000 GenomesAlleleFrequency	TDT^2^	Pvalue
1	*ZNF77*	C>T, Q100X	6	C/T: 17; C/C: 133	T: 0.057	T: 0.022	17 t/7 ut	0.032
2	*KNG1*	C>T, R412X	3	C/T: 10; C/C: 140	T: 0.033	T: 0.015	10 t/11 ut	ns
3	*MMP8*	C>T, Q450X	2	C/T: 8; C/C: 142	T: 0.027	T: 0.011	8 t/5 ut	ns
4	*STARD6*	C>T, R19X	2	TT: 1; C/T: 10; C/C: 139	T: 0.04	T: 0.013	12 t/10 ut	ns
5	*C14orf105*	C>T, Q183X	2	C/T: 8; T/T: 1, C/C: 141	T: 0.033	T:0.018	10 t/8 ut	ns
6	*FAM81B*	C>T, Q144X	2	C/T: 6; C/C: 144	T: 0.02	T: 0.018	6 t/8 ut	ns
7	C11orf40	G>A, W32X	2	A/A: 1; G/A: 19; G/G: 130	A:0.07	A: 0.025	19 t/8 ut	0.026

1 N = number of patients with the variant out of 19; TDT = transmission disequilibrium test applied to 150 probands and their parents; ns = notsignificant.

2 t = transmitted; ut = untransmitted.

### Mutation Analysis of Seven Candidate Genes in the FMS Cohort

We tested the 7 genes in **Table 2** as potential candidate genes by direct sequence analysis of the genes in the 150 FMS probands plus their parents. This analysis revealed complete concordance with the exome sequencing data of the original 19 probands and provided sufficient information to allow transmission analysis of the entire 450 samples in the cohort for all seven genes. Based on this analysis, C11orf40 and *ZNF77* emerged as significantly associated genes in the TDT (**Table 2**). In the transmission analysis, the parents serve as the “controls,” in that transmission of heterozygous rare alleles from parent to offspring should occur at a probability of 0.5 if neutral and >0.5 if associated with FMS. Thus, 2 of the 7 genes analyzed yielded potential associations with the FMS cohort. In addition, we expanded our analysis of the *MEFV* missense mutations from 100 probands and their parents to 150 trios. The increased numbers gave a p value of 0.0030 for significance of FMS association by TDT, compared with our original value of 0.0085 [14]. We also noted that *MEFV* disease-associated SNPs appeared in the group of 905 SNPs described in the previous results section, representing both missense and nonsense variants (**Table S2**).

### C11orf40 (Chromosome 11 Open Reading Frame 40)

The nonsense mutation, W32X, was identified in the C11orf40 gene in 2/19 FMS probands by exome sequencing. Directed sequencing of the additional 131 FMS probands revealed that 17 additional patients were heterozygous and one patient homozygous for this nonsense mutation (W32X, **Table S3**, **Figure S1**). Twenty out of 150 probands (13.3%) carried the W32X variant (FMS allele frequency of 7%, **Table 2**), compared with the 2.5% frequency cutoff in the May 2011 version of the 1000 Genomes database used in filtering step 4 (see above). We tested the W32X variant for preferential transmission using the exact binomial version of the TDT, as described in Methods. In 19 of these 27 events (independent under the null hypothesis), the mutant allele was transmitted to the affected offspring (p = 0.026) (**Table 2** **, Table S3**). Thus, we concluded that the mutant allele encoding a truncating W32X mutation for the C11orf40 gene was associated with FMS.

The C11orf40 gene located on chromosome 11p15.4 has four exons and encodes a putative 217 amino acid protein. Tissue analysis of its gene expression showed that C11orf40 was expressed in normal human tissues including brain, heart, lung, ovary and others, with a very high level of expression in monocytes (**Figure S2**). The function of the C11orf40 gene is unknown, and it has no homology (<10%) to other genes in the human genome.

### *ZNF77* (Zinc Finger Protein 77)

For the *ZNF77* gene, 17 FMS probands were heterozygous for the nonsense mutation, Q100X, in the 150 patient cohort (11.3%). Directed sequencing revealed 24 heterozygous parents that might have transmitted the variant or not **(Figure S1, Table S4)**. Transmission analysis, again using the exact binomial version of the TDT, revealed that the mutant allele was transmitted to the affected offspring in 17 of 24 independent events (**Table 2**, p = 0.032). Thus, this nonsense mutant allele of the *ZNF77* gene was also associated with FMS.

The *ZNF77* gene, located on chromosome 19p13.3, contains four exons encoding a 545 amino acid zinc finger protein. Serial analysis of gene expression revealed that *ZNF77* was expressed in all normal human tissues (data not shown). Although zinc finger proteins are often involved in transcriptional regulation, the functions of most of the *ZNF* genes, including *ZNF77*, are unknown.

### C11orf40 and *ZNF77* Genotype Correlation with Chemokine and Cytokine Plasma Levels

**Figure 1:**
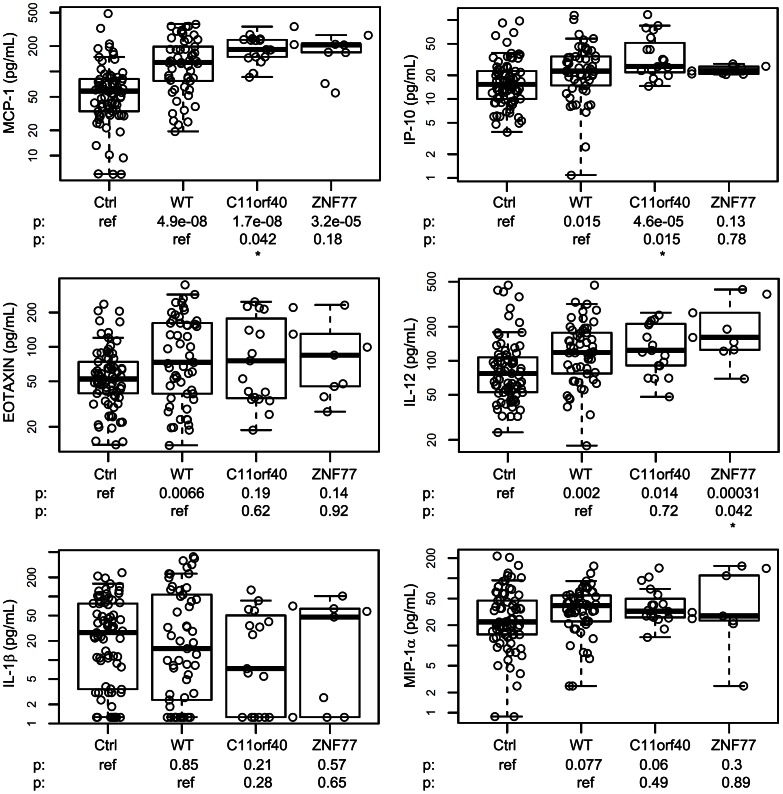
Plasma chemokine/cytokine levelsin FMS patients with C11orf40 or *ZNF77* mutations. Ctrl: unrelated healthy controls, n = 77 (female = 48 and male = 29). WT: FMS patients with wild type (non-variant) C11orf40 and *ZNF77*, n = 53 (female = 45 and male = 8). C11orf40: FMS patients with C11orf40 mutation W32X, n = 20 (female = 13 and male = 7). ZNF77: FMS patients with *ZNF77* mutation Q100X, n = 9 (female = 7 and male = 2). P values in the first row are in comparison with unrelated healthy controls; P values in the second row are in comparison with wild type (non-variant) C11orf40 and/or *ZNF77* genes.Discovery of Potential New Gene Variants and Inflammatory Cytokine Associations with Fibromyalgia Syndrome by Whole Exome Sequencing.

Since we had previously shown that both patients and their parents had high plasma levels of MCP-1, IP-10, IL-12 and eotaxin compared with unrelated controls [16], we reanalyzed our cytokine data to determine if any of these plasma levels were correlated with the C11orf40 and *ZNF77* genotypes. Of the 25 cytokines analyzed, only four showed a significant increase comparing patients to controls. MCP-1 (p = 0.042) and IP-10 (0.015) were the only two with a further difference between patients with the mutation and those without. Patients with the W32X mutation had significantly higher MCP-1 and IP-10 levels compared with patients without the mutation or non-FMS controls (**Figure 1**).

**Figure 2:**
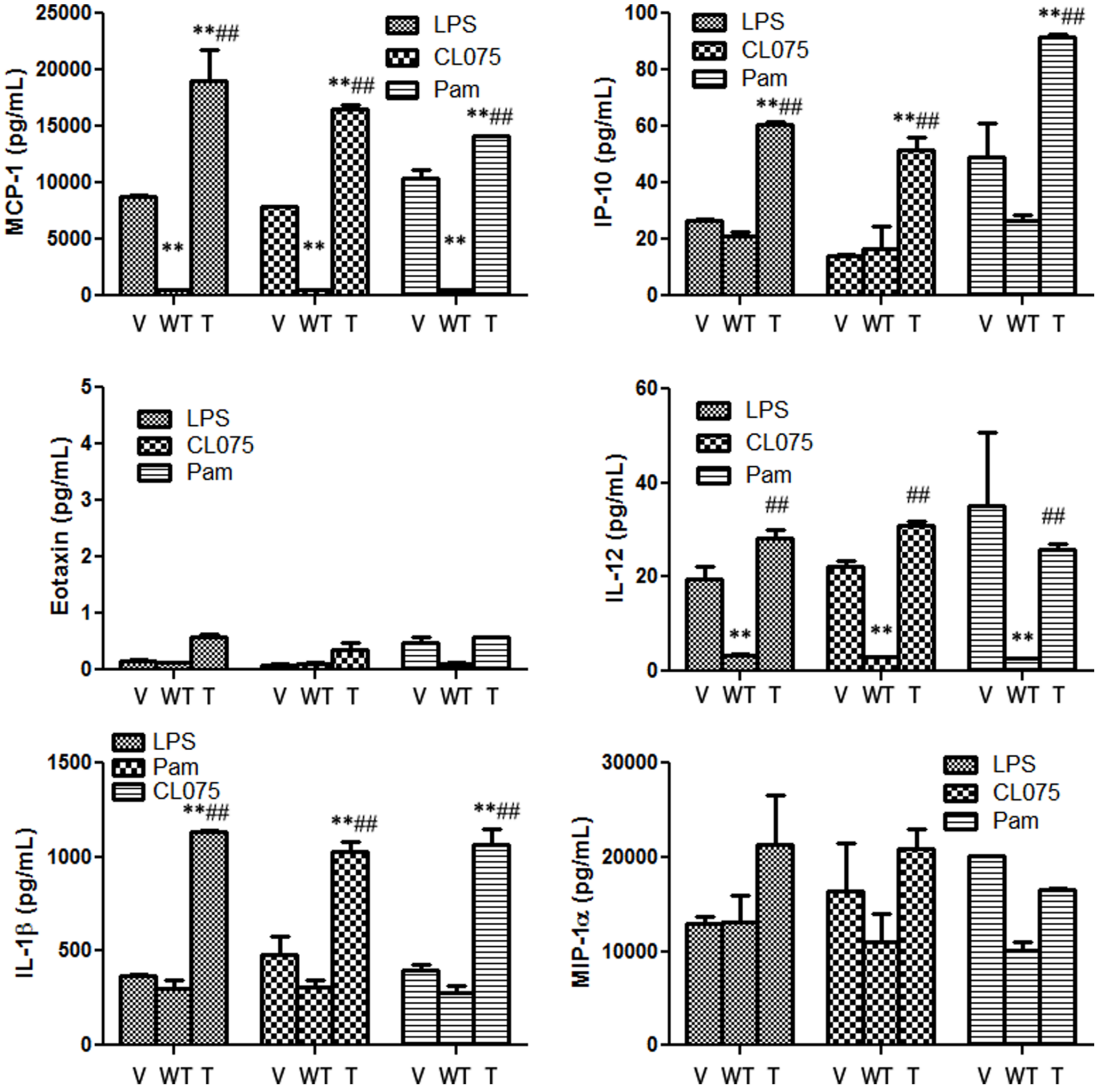
Cytokine secretion of TLR-ligand-treated monocytes transfected with wild type or mutated C11orf40 gene. Human monocytes were transfected with empty vector (V),wild type C11orf40 (WT) or the W32X truncated mutant gene (T) and challenged with three TLR ligands, LPS (TLR4), CL075 (TLR7/8), and Pam3CSK (TLR 1/2), followed by measurement of secreted cytokine 24 hrs. later. P values are for comparison to vector control (**, P = 0.005) or WT (^##^, P = 0.005).

Since C11orf40 was mainly expressed in monocytes (**Figure S2**) and MCP-1 is a key cytokine produced by monocytes, we transfected either the wild type or mutated gene into freshly isolated monocytes and measured cytokine levels 24 h after stimulation with three toll-like receptor ligands, LPS (TLR4), CLO75 (TLR7/8) and Pam3CSK (TLR1/2). The results demonstrated that transfection with the mutant gene resulted in a 2-fold increase in MCP-1 production vs. vector control transfectants for all three treatments (**Figure 2**). Interestingly, the transfectants with the wild type gene *suppressed* MCP-1 production to background levels, suggesting that the wild type gene down-regulates MCP-1 secretion. The results for IP-10 were similar, in that the mutant gene elevated IP-10 levels. In contrast with MCP-1, however, the wild type gene had no effect compared with vector controls, indicating that C11orf40 suppression of MCP-1 was specific. We conclude from this preliminary analysis that the C11orf40 gene has a role in MCP-1 and IP-10 secretion and that the presence of the mutant gene may affect secretion of MCP-1 in both isolated monocytes and, perhaps, in patients with FMS. Further functional studies are required to dissect the relatively unstudied pathway for MCP-1 secretion in monocytes.

Although fewer patient data were available for *ZNF77*, a statistically significant cytokine result was also seen for this gene. Among the 25 cytokines analyzed, IL-12 plasma levels (p = 0.042) were elevated in patients with the mutant gene (**Figure 1**).

## Discussion

Exome sequencing of 19 FMS patients, followed by filtering of the over 80,000 SNPs per patient according to 3 principal criteria (occurred in 2/19 probands, was a nonsense mutation, and had an AAF of 2.5% or less in the 1000 Genomes database), led to the selection of 7 candidate genes. Validation by direct sequencing in the entire cohort of 150 trios and TDT analysis identified two possible disease susceptibility genes in a subset of about 25% of FMS probands. Combined with our analysis of *MEFV* SNPs in the same cohort, we found a total of 35% of FMS probands carried one or more mutations in these three genes.

Often, one of the hallmarks of inheriting disease-predisposing gene variants is early onset of disease. Among the 150 patients in our cohort, the age distribution for symptom onset was quite broad–from 10 to 68 years of age (**Table 1**). However, among 9 patients with at least two implicated variants of Cllorf40, *ZNF77*, and *MEFV*, 3 showed symptom onset at age 10 and one before age 18–all at the extremely young end of the age of symptom onset distribution. Although exome sequencing can be considered as an unbiased method for identifying new candidate genes in a disease, our filtering strategy, in drastically reducing the variants taken through TDT and subsequent functional analysis, has left the strong possibility that other classes of genetic variants we have observed may also be implicated in FMS. However, our present study design lacks the power to examine this likely eventuality. Most notable among these classes of variants are the thousands of unique variants in our 19 probands, as well as the filtered set of 905 variants that include missense mutations, in addition to the nonsense mutations we more fully characterized in this report. Larger case-control studies, in which directed sequencing of candidates identified from this discovery tabulation is performed, will be the next step in completing the analysis of FMS genetic susceptibility.

Although our SNP filtering strategy dramatically reduced the number of gene variants that we ultimately analyzed further, the method was completely unbiased with regard to mechanism and functional categories. After filtering the variants down to 7, TDT analysis separated these into two groups, two genes with evidence of differential transmission and five genes with no such evidence. As it transpired, the two candidates that emerged from our study, C11orf40 and *ZNF77*, were not previously assigned functions or functional pathways. However, these two genes were further shown to be associated with high levels of specific cytokines, with a dramatic and highly significant elevation compared to normal controls. If our hypothesis was correct that FMS has, at least in part, an inflammatory basis, then a positive correlation with our previous cytokine expression results was to be expected. Indeed, this was the result that we obtained for both C11orf40 and *ZNF77*. Furthermore, when the wild type C11orf40 gene was transfected into monocytes, TLR-stimulated secretion of MCP-1 and IP-10 was reduced to background levels, while transfection of the mutated gene elevated secretion of these cytokines two-fold over that of vector-transfected cells. The notion that 3 of 4 FMS-elevated cytokines are further elevated by variants at one of two genes can be formalized as the chance of 3 or more spurious findings at the 0.05 level under the global null hypothesis (0.0058). Combined with our previous study that demonstrated a positive TDT test for *MEFV* gene variants and an association with elevated IL-1β levels, we now have identified 3 potential genes associated with the risk of FMS, associated with elevated levels of inflammatory cytokines and accounting for 35% of our FMS cohort.

## Supporting Information

Figure S1 Example of sequence analysis of proband with the C11orf40 mutation or the *ZNF77* mutation. Upper: Examples of heterozygotes for the mutation G>A or homozygotes for G (wild type) for C11orf40 at position Chr11_4598956. Lower: Examples of heterozygotes for the mutation T>C or homozygotes for T (wild type) for *ZNF77* at position chr19_2936535.
(DOCX)

Figure S2 Tissue and cell expression analysis of C11orf40 gene. A, B: PCR analysis for C11orf40 in a variety of tissues compared with housekeeping gene, GAPDH. **C**: Quantitative PCR analysis of C11orf40 expression in human monocytes and 3 human myeloid cell lines, HL60, THP1, and U937, normalized to GAPDH.
(DOCX)

Table S1 Summary of exome sequencing on 19 probands with FMS. Nineteen probands (FMS512 was sequenced twice) were subjected to exome sequence analysis. Total reads, total mapped reads, total yields, the percent coding exon >5× coverage, total SNPs and concordance with dbsNP134 are reported.
(DOCX)

Table S2 SNPs sorted for present in 2/19 patiemts and in 2.5% or less of the genome 1000 dbase. The SNP ID, gene symbol, amino acid change, allele frequency, frequency in the genome 1000 dbase, and the number of patients with the SNP are reported.
(DOCX)

Table S3 Transmission analysis for mutation W32X of the C11orf40 gene. The genotypes (heterozygous or homozygous) for proband, mother and father are reported together with the analysis of transmitted (t(B)) or untransmitted (ut(C)) from parent to proband. P = 0.026.
(DOCX)

Table S4 Transmission analysis for Q100X of ZNF77 gene. The genotypes (heterozygous or homozygous) for proband, mother and father are reported together with the analysis of transmitted (t(B)) or untransmitted (ut(C)) from parent to proband. P = 0.032.
(DOCX)

## References

[1] Wolfe F, Cathey MA (1990) Assessment of functional ability in patients with fibromyalgia. Arch Intern Med 150: 460. 2302023

[2] Wolfe F, Ross K, Anderson J, Russell IJ, Hebert L (1995) The prevalence and characteristics of fibromyalgia in the general population. Arthritis Rheum 38: 19–28. 7818567 10.1002/art.1780380104

[3] Buskila D, Neumann L, Sibirski D, Shvartzman P (1997) Awareness of diagnostic and clinical features of fibromyalgia among family physicians. Fam Pract 14: 238–241. 9201499 10.1093/fampra/14.3.238

[4] Arnold LM, Hudson JI, Hess EV, Ware AE, Fritz DA, et al (2004) Family study of fibromyalgia. Arthritis Rheum 50: 944–952. 15022338 10.1002/art.20042

[5] Kato K, Sullivan PF, Evengard B, Pedersen NL (2006) Chronic widespread pain and its comorbidities: a population-based study. Arch Intern Med 166: 1649–1654. 16908799 10.1001/archinte.166.15.1649

[6] Bondy B, Spaeth M, Offenbaecher M, Glatzeder K, Stratz T, et al (1999) The T102C polymorphism of the 5-HT2A-receptor gene in fibromyalgia. Neurobiol Dis 6: 433–439. 10527809 10.1006/nbdi.1999.0262

[7] Gursoy S, Erdal E, Herken H, Madenci E, Alasehirli B (2001) Association of T102C polymorphism of the 5-HT2A receptor gene with psychiatric status in fibromyalgia syndrome. Rheumatol Int 21: 58–61. 11732859 10.1007/s002960100130

[8] Buskila D, Cohen H, Neumann L, Ebstein RP (2004) An association between fibromyalgia and the dopamine D4 receptor exon III repeat polymorphism and relationship to novelty seeking personality traits. Mol Psychiatry 9: 730–731. 15052273 10.1038/sj.mp.4001506

[9] Buskila D, Sarzi-Puttini P, Ablin JN (2007) The genetics of fibromyalgia syndrome. Pharmacogenomics 8: 67–74. 17187510 10.2217/14622416.8.1.67

[10] Smith SB, Maixner DW, Fillingim RB, Slade G, Gracely RH, et al (2012) Large candidate gene association study reveals genetic risk factors and therapeutic targets for fibromyalgia. Arthritis and rheumatism 64: 584–593. 21905019 10.1002/art.33338 PMC3237946

[11] Berkun Y, Eisenstein E, Ben-Chetrit E (2012) FMF - clinical features, new treatments and the role of genetic modifiers: a critical digest of the 2010–2012 literature. Clinical and experimental rheumatology. 23009752

[12] Romano TJ (1988) Coexistence of irritable bowel syndrome and fibromyalgia. The West Virginia medical journal 84: 16–18. 3422770

[13] Sperber AD, Akiva S, Leshno M, Halpern Z, Buskila D (2011) Validation of New Symptom-Based Fibromyalgia Criteria for Irritable Bowel Syndrome Co-morbidity Studies. Journal of neurogastroenterology and motility 17: 67–72. 21369494 10.5056/jnm.2011.17.1.67 PMC3042222

[14] Feng J, Zhang Z, Li W, Shen X, Song W, et al (2009) Missense mutations in the MEFV gene are associated with fibromyalgia syndrome and correlate with elevated IL-1beta plasma levels. PLoS One 4: e8480. 20041150 10.1371/journal.pone.0008480 PMC2794536

[15] Wolfe F, Smythe HA, Yunus MB, Bennett RM, Bombardier C, et al (1990) The American College of Rheumatology 1990 Criteria for the Classification of Fibromyalgia. Report of the Multicenter Criteria Committee. Arthritis Rheum 33: 160–172. 2306288 10.1002/art.1780330203

[16] Zhang Z, Cherryholmes G, Mao A, Marek C, Longmate J, et al (2008) High plasma levels of MCP-1 and eotaxin provide evidence for an immunological basis of fibromyalgia. Exp Biol Med (Maywood) 233: 1171–1180. 18535166 10.3181/0712-RM-328

[17] Spielman RS, McGinnis RE, Ewens WJ (1993) Transmission test for linkage disequilibrium: the insulin gene region and insulin-dependent diabetes mellitus (IDDM). Am J Hum Genet 52: 506–516. 8447318 PMC1682161

