# Supplementary material for: Discovery of Potential New Gene Variants and Inflammatory Cytokine Associations with Fibromyalgia Syndrome by Whole Exome Sequencing
Source: PLoS One. 2013 Jun 10;8(6):e65033. doi: 10.1371/journal.pone.0065033 (PMC3677902; doi:10.1371/journal.pone.0065033)

**Figure S1. Example of sequence analysis of proband with the C11orf40 mutation or the *ZNF77* mutation.** Upper: Examples of heterozygotes for the mutation G>A or homozygotes for G (wild type) for C11orf40 at position Chr11_4598956. Lower: Examples of heterozygotes for the mutation T>C or homozygotes for T (wild type) for *ZNF77* at position chr19_2936535.


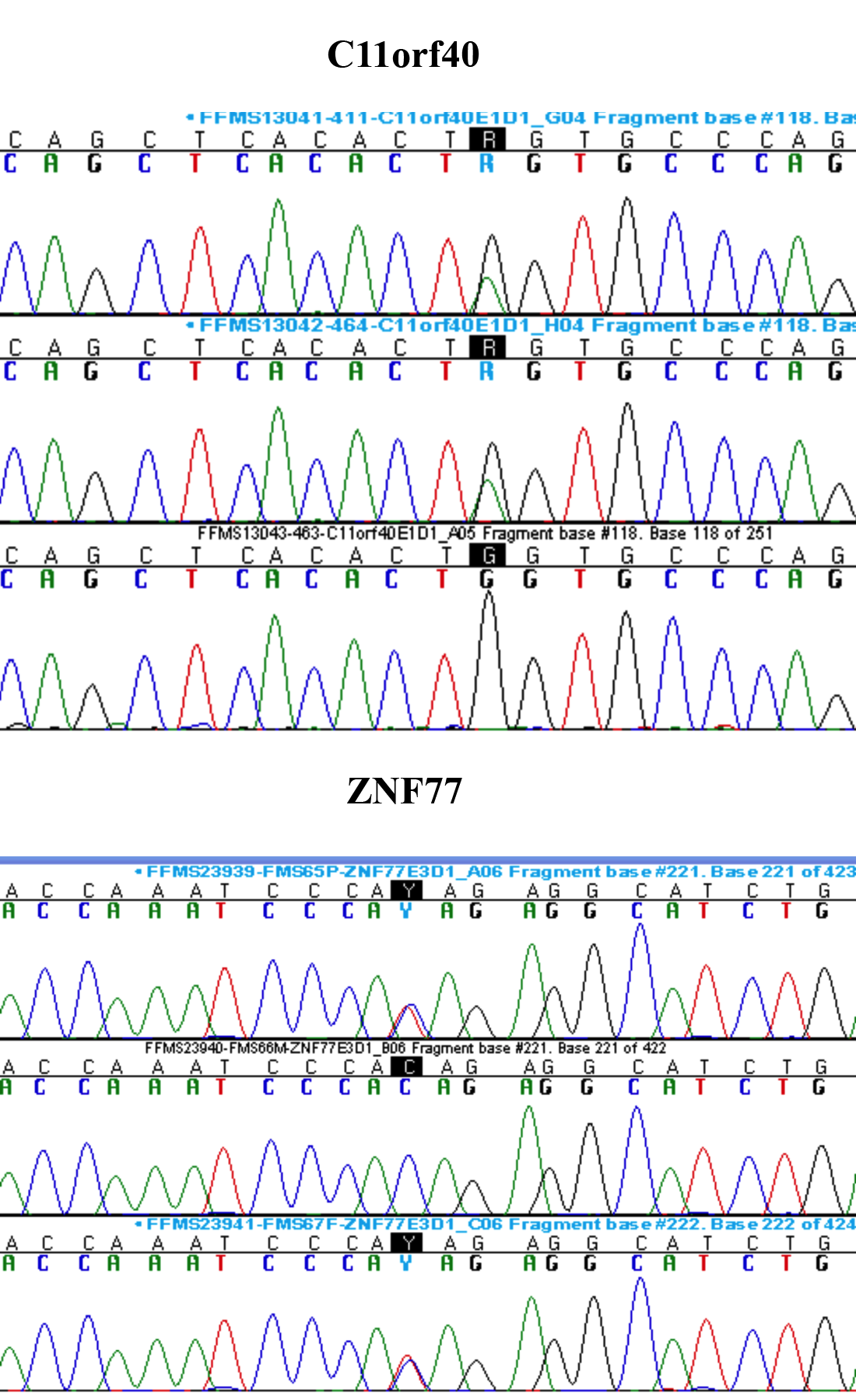

Supplement: Figure S1 — Example of sequence analysis of proband with the C11orf40 mutation or the ZNF77 mutation. Upper: Examples of heterozygotes for the mutation G>A or homozygotes for G (wild type) for C11orf40 at position Chr11_4598956. Lower: Examples of heterozygotes for the mutation T>C or homozygotes for T (wild type) for ZNF77 at position chr19_2936535. (DOCX) [file pone.0065033.s001.docx]
