# Supplementary material for: Discovery of Potential New Gene Variants and Inflammatory Cytokine Associations with Fibromyalgia Syndrome by Whole Exome Sequencing
Source: PLoS One. 2013 Jun 10;8(6):e65033. doi: 10.1371/journal.pone.0065033 (PMC3677902; doi:10.1371/journal.pone.0065033)

**Figure S2. Tissue and cell expression analysis of C11orf40 gene. A, B:** PCR analysis for C11orf40 in a variety of tissues compared with housekeeping gene, *GAPDH*. **C:** Quantitative PCR analysis of C11orf40 expression in human monocytes and 3 human myeloid cell lines, HL60, THP1, and U937, normalized to GAPDH.


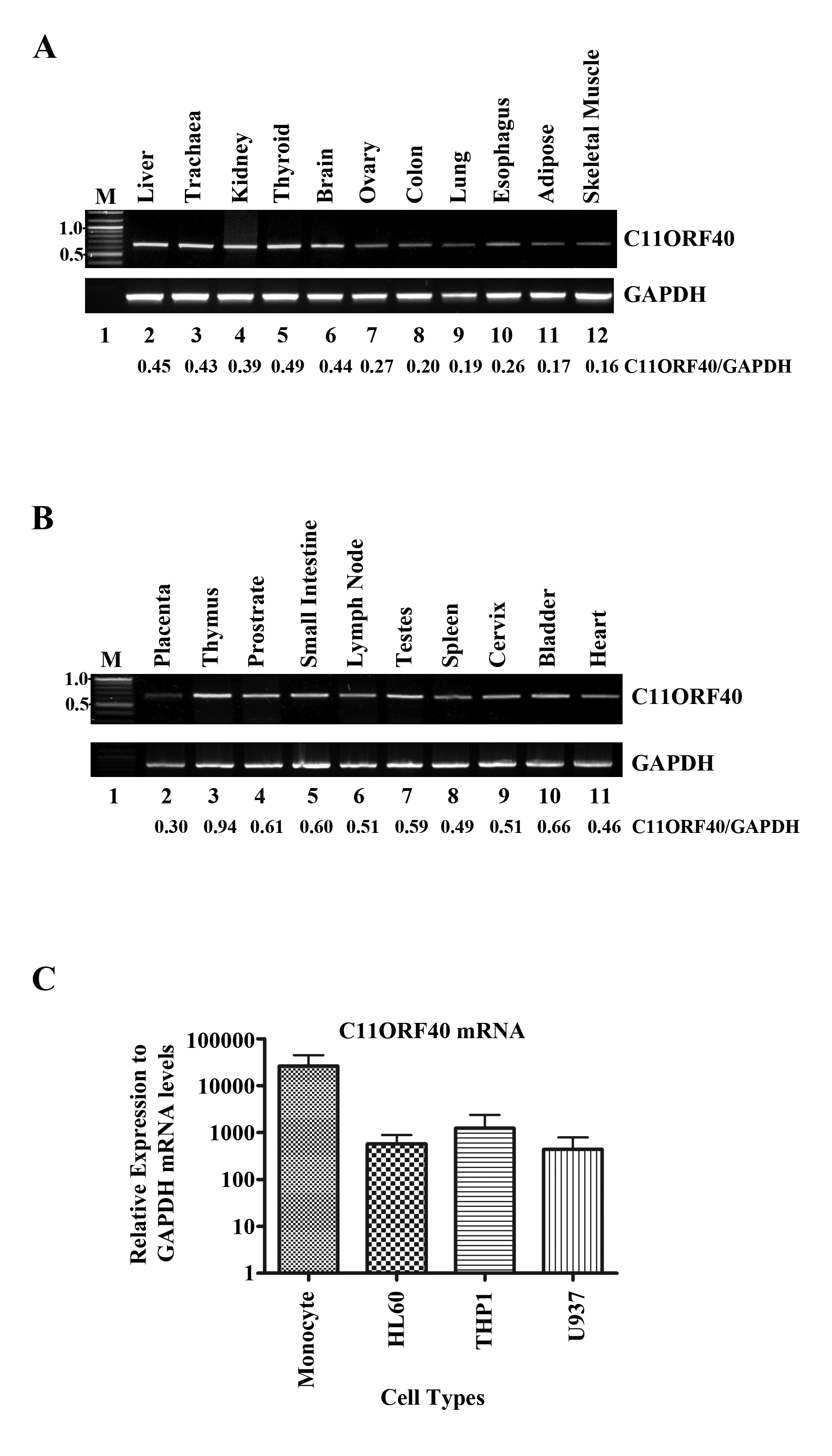

Supplement: Figure S2 — Tissue and cell expression analysis of C11orf40 gene. A, B: PCR analysis for C11orf40 in a variety of tissues compared with housekeeping gene, GAPDH. C: Quantitative PCR analysis of C11orf40 expression in human monocytes and 3 human myeloid cell lines, HL60, THP1, and U937, normalized to GAPDH. (DOCX) [file pone.0065033.s002.docx]
