# Supplementary material for: Discovery of Potential New Gene Variants and Inflammatory Cytokine Associations with Fibromyalgia Syndrome by Whole Exome Sequencing
Source: PLoS One. 2013 Jun 10;8(6):e65033. doi: 10.1371/journal.pone.0065033 (PMC3677902; doi:10.1371/journal.pone.0065033)
Supplement: Table S1 — Summary of exome sequencing on 19 probands with FMS. Nineteen probands (FMS512 was sequenced twice) were subjected to exome sequence analysis. Total reads, total mapped reads, total yields, the percent coding exon >5× coverage, total SNPs and concordance with dbsNP134 are reported. (DOCX) [file pone.0065033.s003.docx]

**Table S1. Summary of exome sequencing on 19 probands with FMS.** Nineteen probands (FMS512 was sequenced twice) were subjected to exome sequence analysis. Total reads, total mapped reads, total yields, the percent coding exon >5X coverage, total SNPs and concordance with dbsNP134 are reported.

| Proband_ID | Total Reads (Millions) | Mapped Reads (Millions) | % Mapped Reads | Total Yields (Gbp) | Coding Exon Coverage (> 5X) | Median Coding Exon Coverage | Total SNP No. | Concordance with dbSNP134 |
| --- | --- | --- | --- | --- | --- | --- | --- | --- |
| FMS104 | 56.2 | 35.7 | 63.50% | 2,856 | 82.4% | 25.3 | 83,352 | 95.50% |
| FMS127 | 55.7 | 39.6 | 71.10% | 3,168 | 80.4% | 36.2 | 45,808 | 97.20% |
| FMS321 | 53 | 37.4 | 70.60% | 2,992 | 80.1% | 34.4 | 52,543 | 96.60% |
| FMS340 | 54.6 | 34.8 | 63.70% | 2,784 | 82.3% | 24.4 | 82,763 | 95.90% |
| FMS45 | 31.7 | 25.8 | 81.40% | 2,064 | 77.3% | 23.3 | 48,375 | 96.80% |
| FMS483 | 99 | 42.5 | 42.90% | 3,400 | 79.4% | 19.9 | 321,466 | 92.80% |
| FMS495 | 53.6 | 41.5 | 77.40% | 3,320 | 77.8% | 31.0 | 59,678 | 96.70% |
| FMS501 | 59.5 | 43.8 | 73.60% | 3,504 | 77.4% | 37.1 | 58,879 | 96.80% |
| FMS51 | 32.3 | 26.6 | 82.40% | 2,128 | 81.2% | 23.4 | 53,613 | 96.50% |
| FMS512 | 63.5 | 44.1 | 69.40% | 3,528 | 81.3% | 40.9 | 48,477 | 96.90% |
| FMS512 | 55.9 | 38 | 68.00% | 3,040 | 77.4% | 35.6 | 56,564 | 96.30% |
| FMS53 | 51.5 | 38.1 | 74.00% | 3,048 | 80.1% | 35.1 | 50,363 | 97.10% |
| FMS540 | 60.3 | 44.8 | 74.30% | 3,584 | 81.2% | 40.9 | 70,195 | 96.00% |
| FMS549 | 59 | 42.1 | 71.40% | 3,368 | 78.0% | 41.2 | 55,266 | 96.60% |
| FMS65 | 127.7 | 30.4 | 23.80% | 2,432 | 78.6% | 26.6 | 102,392 | 94.90% |
| FMS68 | 132.8 | 59.8 | 45.00% | 4,784 | 83.5% | 44.5 | 107,252 | 97.10% |
| FMS76 | 133.1 | 63.6 | 47.80% | 5,088 | 85.0% | 51.4 | 105,526 | 97.10% |
| FMS82 | 49.9 | 28.8 | 57.70% | 2,304 | 68.4% | 15.9 | 75,653 | 95.40% |
| FMS97 | 53.6 | 32.1 | 59.90% | 2,568 | 81.7% | 22.1 | 92,751 | 95.70% |
| FMS99 | 53.8 | 32.9 | 61.20% | 2,632 | 81.1% | 21.9 | 83,854 | 95.30% |
