# Supplementary material for: Discovery of Potential New Gene Variants and Inflammatory Cytokine Associations with Fibromyalgia Syndrome by Whole Exome Sequencing
Source: PLoS One. 2013 Jun 10;8(6):e65033. doi: 10.1371/journal.pone.0065033 (PMC3677902; doi:10.1371/journal.pone.0065033)
Supplement: Table S2 — SNPs sorted for present in 2/19 patiemts and in 2.5% or less of the genome 1000 dbase. The SNP ID, gene symbol, amino acid change, allele frequency, frequency in the genome 1000 dbase, and the number of patients with the SNP are reported. (DOCX) [file pone.0065033.s004.docx]

**Table S2. SNPs sorted for present in 2/19 patients and in 2.5% or less of the genome 1000 dbase^1^.**

| ID | Gene Symbol | AA Change | Allele  Freq | 1000  Genome Freq | n |
| --- | --- | --- | --- | --- | --- |
| chr1_31896645_G_T | SERINC2 | GLY-TRP | 0.056 | 0.001 | 2 |
| chr2_86716518_G_A | KDM3A | VAL-ILE | 0.053 | 0.001 | 2 |
| chr2_112786034_C_T | MERTK | ARG-TRP | 0.053 | 0.001 | 2 |
| chr2_163361158_T_A | KCNH7 | ASN-ILE | 0.056 | 0.001 | 2 |
| chr3_124418847_G_A | KALRN | GLU-LYS | 0.053 | 0.001 | 2 |
| chr3_148459552_G_T | AGTR1 | ALA-SER | 0.063 | 0.001 | 2 |
| chr3_186395400_G_A | HRG | GLY-ARG | 0.053 | 0.001 | 2 |
| chr4_126329821_A_G | FAT4 | TYR-CYS | 0.053 | 0.001 | 2 |
| chr5_43656074_C_T | NNT | THR-MET | 0.053 | 0.001 | 2 |
| chr5_55237432_A_C | IL6ST | ASP-GLU | 0.053 | 0.001 | 2 |
| chr5_134345062_A_G | CATSPER3 | ASP-GLY | 0.053 | 0.001 | 2 |
| chr5_138378394_G_A | SIL1 | THR-ILE | 0.053 | 0.001 | 2 |
| chr6_106968250_G_T | AIM1 | SER-ILE | 0.053 | 0.001 | 2 |
| chr7_102575018_T_A | LRRC17,FBXL13 | LEU-MET | 0.053 | 0.001 | 2 |
| chr8_95201468_G_A | CDH17 | PRO-SER | 0.079 | 0.001 | 2 |
| chr10_24817027_G_T | KIAA1217 | VAL-LEU | 0.059 | 0.001 | 2 |
| chr10_45799452_C_T | OR13A1 | ARG-GLN | 0.094 | 0.001 | 2 |
| chr10_50678884_T_G | ERCC6 | GLN-PRO | 0.053 | 0.001 | 2 |
| chr10_50690906_G_A | ERCC6 | ARG-CYS | 0.063 | 0.001 | 2 |
| chr10_121182694_C_T | GRK5 | ALA-VAL | 0.053 | 0.001 | 2 |
| chr11_7063792_G_A | NLRP14 | VAL-MET | 0.053 | 0.001 | 2 |
| chr11_57947505_G_C | OR9Q1 | VAL-LEU | 0.053 | 0.001 | 2 |
| chr12_1943732_G_A | CACNA2D4,LRTM2 | VAL-ILE | 0.053 | 0.001 | 2 |
| chr12_95456418_C_T | NR2C1 | GLY-SER | 0.053 | 0.001 | 2 |
| chr12_112184086_G_A | ACAD10 | GLU-LYS | 0.053 | 0.001 | 2 |
| chr13_45589616_C_G | KIAA1704 | PRO-ARG | 0.056 | 0.001 | 2 |
| chr13_45913651_G_C | TPT1 | ALA-GLY | 0.105 | 0.001 | 4 |
| chr14_23467755_G_A | C14orf93 | ARG-TRP | 0.053 | 0.001 | 2 |
| chr15_86287031_C_A | AKAP13 | ASN-LYS | 0.118 | 0.001 | 4 |
| chr15_89825056_A_G | FANCI | MET-VAL | 0.105 | 0.001 | 3 |
| chr17_8052915_C_T | PER1 | VAL-ILE | 0.063 | 0.001 | 2 |
| chr17_39728000_C_T | KRT9 | SER-ASN | 0.053 | 0.001 | 2 |
| chr19_18119848_G_A | ARRDC2 | ARG-GLN | 0.053 | 0.001 | 2 |
| chr19_19030611_G_A | DDX49 | GLY-SER | 0.059 | 0.001 | 2 |
| chr20_60884852_G_A | LAMA5 | ALA-VAL | 0.053 | 0.001 | 2 |
| chr1_32145693_C_T | COL16A1 | GLY-ARG | 0.056 | 0.002 | 2 |
| chr1_147131553_T_C | ACP6 | ASP-GLY | 0.053 | 0.002 | 2 |
| chr1_151140732_G_A | SCNM1,TNFAIP8L2 | GLU-LYS | 0.053 | 0.002 | 2 |
| chr1_151143016_T_C | TMOD4 | MET-VAL | 0.053 | 0.002 | 2 |
| chr1_151867657_G_C | THEM4 | SER-CYS | 0.088 | 0.002 | 3 |
| chr1_182369105_C_T | TEDDM1 | MET-ILE | 0.053 | 0.002 | 2 |
| chr2_68729939_A_G | APLF | TYR-CYS | 0.056 | 0.002 | 2 |
| chr2_170350279_A_G | BBS5 | ASN-SER | 0.067 | 0.002 | 2 |
| chr2_228173636_A_G | COL4A3 | GLN-ARG | 0.053 | 0.002 | 2 |
| chr3_49935526_T_G | MST1R | GLN-PRO | 0.053 | 0.002 | 2 |
| chr3_120495353_C_T | GTF2E1 | SER-PHE | 0.053 | 0.002 | 2 |
| chr4_54218924_C_A | SCFD2 | GLY-VAL | 0.059 | 0.002 | 2 |
| chr4_57829669_G_A | C4orf14 | PRO-SER | 0.053 | 0.002 | 2 |
| chr4_126372555_A_G | FAT4 | ILE-VAL | 0.056 | 0.002 | 2 |
| chr4_169060675_C_T | ANXA10 | ARG-CYS | 0.056 | 0.002 | 2 |
| chr6_30520367_C_T | GNL1 | GLU-LYS | 0.053 | 0.002 | 2 |
| chr6_44361309_G_A | CDC5L | none | 0.053 | 0.002 | 2 |
| chr6_74440124_G_A | CD109 | ASP-ASN | 0.053 | 0.002 | 2 |
| chr7_27196116_C_T | HOXA7 | GLY-ARG | 0.053 | 0.002 | 2 |
| chr7_91700267_T_C | AKAP9 | SER-PRO | 0.053 | 0.002 | 2 |
| chr7_117267812_T_G | CFTR | SER-ARG | 0.053 | 0.002 | 2 |
| chr8_133899575_G_A | TG | GLY-ASP | 0.056 | 0.002 | 2 |
| chr9_98011497_G_A | FANCC | SER-PHE | 0.053 | 0.002 | 2 |
| chr11_65638718_C_T | EFEMP2 | GLY-SER | 0.053 | 0.002 | 2 |
| chr12_15777273_C_T | EPS8 | ALA-THR | 0.053 | 0.002 | 2 |
| chr12_51451867_G_A | LETMD1 | ARG-GLN | 0.053 | 0.002 | 2 |
| chr12_64814251_G_A | XPOT | GLU-LYS | 0.056 | 0.002 | 2 |
| chr12_129299435_C_T | SLC15A4 | GLY-SER | 0.053 | 0.002 | 2 |
| chr12_132401539_C_T | ULK1 | ALA-VAL | 0.053 | 0.002 | 2 |
| chr14_23532193_T_C | ACIN1 | GLN-ARG | 0.053 | 0.002 | 2 |
| chr15_31197015_T_G | FAN1 | MET-ARG | 0.056 | 0.002 | 2 |
| chr16_10788550_C_T | TEKT5 | VAL-ILE | 0.053 | 0.002 | 2 |
| chr17_8413233_G_A | MYH10 | ALA-VAL | 0.053 | 0.002 | 2 |
| chr18_9887582_C_A | TXNDC2 | PRO-HIS | 0.053 | 0.002 | 2 |
| chr19_35633635_C_T | FXYD1 | ARG-CYS | 0.056 | 0.002 | 2 |
| chr20_1961298_T_G | PDYN | MET-LEU | 0.053 | 0.002 | 2 |
| chr20_16360240_G_A | KIF16B | ARG-TRP | 0.053 | 0.002 | 2 |
| chr20_62597996_T_C | ZNF512B | ARG-GLY | 0.059 | 0.002 | 2 |
| chr22_45312474_C_T | PHF21B | VAL-ILE | 0.056 | 0.002 | 2 |
| chr22_45723898_G_A | FAM118A | ARG-GLN | 0.250 | 0.002 | 8 |
| chr1_39763365_G_T | MACF1 | CYS-PHE | 0.053 | 0.003 | 2 |
| chr1_114255975_G_T | PHTF1 | GLN-LYS | 0.079 | 0.003 | 2 |
| chr2_70441562_T_C | TIA1 | GLN-ARG | 0.079 | 0.003 | 3 |
| chr2_238275569_T_C | COL6A3 | LYS-ARG | 0.053 | 0.003 | 2 |
| chr5_56778235_A_C | ACTBL2 | ASP-GLU | 0.053 | 0.003 | 2 |
| chr5_112399751_C_G | MCC | GLY-ARG | 0.053 | 0.003 | 2 |
| chr5_176813433_C_T | SLC34A1 | ALA-VAL | 0.053 | 0.003 | 2 |
| chr6_32159931_G_C | GPSM3 | HIS-ASP | 0.067 | 0.003 | 2 |
| chr6_71377781_C_A | SMAP1 | LEU-ILE | 0.053 | 0.003 | 2 |
| chr8_17500162_T_A | PDGFRL | ILE-ASN | 0.053 | 0.003 | 2 |
| chr8_145773779_C_T | ARHGAP39 | GLY-SER | 0.056 | 0.003 | 2 |
| chr9_35042268_A_G | C9orf131 | GLU-GLY | 0.079 | 0.003 | 3 |
| chr10_3162130_A_G | PFKP | GLU-GLY | 0.056 | 0.003 | 2 |
| chr10_14816378_G_C | FAM107B | HIS-GLN | 0.053 | 0.003 | 2 |
| chr11_55861523_T_C | OR8I2 | VAL-ALA | 0.053 | 0.003 | 2 |
| chr11_56230010_G_T | OR5M9 | LEU-MET | 0.053 | 0.003 | 2 |
| chr12_96181085_G_A | NTN4 | ARG-TRP | 0.053 | 0.003 | 2 |
| chr13_31729776_T_G | HSPH1 | ASN-HIS | 0.053 | 0.003 | 2 |
| chr13_31848718_G_A | B3GALTL | VAL-MET | 0.053 | 0.003 | 2 |
| chr14_45606290_C_T | FANCM | THR-ILE | 0.056 | 0.003 | 2 |
| chr14_105416323_T_C | AHNAK2 | LYS-ARG | 0.313 | 0.003 | 5 |
| chr19_5455543_G_C | ZNRF4 | SER-THR | 0.053 | 0.003 | 2 |
| chr19_13084333_G_A | DAND5 | ARG-HIS | 0.053 | 0.003 | 2 |
| chr19_40424269_C_T | FCGBP | GLY-GLU | 0.059 | 0.003 | 2 |
| chr19_52618471_C_T | ZNF616 | ARG-LYS | 0.053 | 0.003 | 2 |
| chr19_56160797_G_A | CCDC106 | ALA-THR | 0.056 | 0.003 | 2 |
| chrX_50658966_G_A | BMP15 | ALA-THR | 0.053 | 0.003 | 2 |
| chrX_128884473_G_A | XPNPEP2 | VAL-ILE | 0.059 | 0.003 | 2 |
| chrX_153594535_G_A | FLNA | THR-MET | 0.079 | 0.003 | 3 |
| chr1_3683159_A_C | CCDC27 | ASN-HIS | 0.053 | 0.004 | 2 |
| chr1_11733846_C_A | FBXO6 | GLN-LYS | 0.053 | 0.004 | 2 |
| chr1_156255448_A_G | TMEM79 | ASP-GLY | 0.059 | 0.004 | 2 |
| chr1_156911718_C_T | ARHGEF11 | MET-ILE | 0.053 | 0.004 | 2 |
| chr1_157771966_T_C | FCRL1 | ILE-VAL | 0.053 | 0.004 | 2 |
| chr1_236721780_C_T | HEATR1 | ARG-HIS | 0.053 | 0.004 | 2 |
| chr1_238050081_T_C | ZP4 | SER-GLY | 0.053 | 0.004 | 2 |
| chr2_73717921_C_G | ALMS1 | ASN-LYS | 0.079 | 0.004 | 2 |
| chr2_144765026_T_C | GTDC1 | MET-VAL | 0.053 | 0.004 | 2 |
| chr2_160994197_C_T | ITGB6 | GLY-ARG | 0.079 | 0.004 | 3 |
| chr2_208988958_T_C | CRYGD | MET-VAL | 0.067 | 0.004 | 2 |
| chr2_241808308_C_A | AGXT | THR-ASN | 0.133 | 0.004 | 4 |
| chr3_119256020_G_A | CD80 | HIS-TYR | 0.053 | 0.004 | 2 |
| chr3_127298917_T_C | TPRA1 | SER-GLY | 0.053 | 0.004 | 2 |
| chr3_132403418_C_T | NPHP3 | ALA-THR | 0.056 | 0.004 | 2 |
| chr4_147561147_C_A | POU4F2 | ASP-GLU | 0.053 | 0.004 | 2 |
| chr5_102894730_C_T | NUDT12 | VAL-ILE | 0.053 | 0.004 | 2 |
| chr5_112903383_T_G | YTHDC2 | ILE-MET | 0.056 | 0.004 | 2 |
| chr5_150921969_C_T | FAT2 | ALA-THR | 0.056 | 0.004 | 2 |
| chr5_150947751_G_A | FAT2 | PRO-SER | 0.056 | 0.004 | 2 |
| chr5_151202476_C_T | GLRA1 | GLY-SER | 0.053 | 0.004 | 2 |
| chr5_162868905_A_C | CCNG1 | ASN-HIS | 0.125 | 0.004 | 2 |
| chr6_54219405_G_C | TINAG | LYS-ASN | 0.063 | 0.004 | 2 |
| chr6_111587204_A_G | KIAA1919 | THR-ALA | 0.053 | 0.004 | 2 |
| chr6_121768751_C_T | GJA1 | ALA-VAL | 0.079 | 0.004 | 3 |
| chr7_157931003_C_T | PTPRN2 | ARG-HIS | 0.056 | 0.004 | 2 |
| chr8_17541943_T_G | MTUS1 | LYS-THR | 0.105 | 0.004 | 4 |
| chr8_20112355_A_G | LZTS1 | LEU-PRO | 0.053 | 0.004 | 2 |
| chr9_20990164_G_A | KIAA1797 | ALA-THR | 0.053 | 0.004 | 2 |
| chr9_88938668_G_A | ZCCHC6 | THR-ILE | 0.079 | 0.004 | 3 |
| chr9_125673614_C_G | ZBTB6 | MET-ILE | 0.056 | 0.004 | 2 |
| chr9_134027138_A_G | NUP214 | ILE-VAL | 0.059 | 0.004 | 2 |
| chr11_4869658_G_T | OR51S1 | PRO-THR | 0.053 | 0.004 | 2 |
| chr11_67275063_C_T | CDK2AP2 | none | 0.053 | 0.004 | 2 |
| chr12_40114781_A_G | C12orf40 | THR-ALA | 0.056 | 0.004 | 2 |
| chr14_20876282_G_A | TEP1 | SER-PHE | 0.079 | 0.004 | 3 |
| chr14_90767609_C_T | C14orf102 | VAL-MET | 0.053 | 0.004 | 2 |
| chr15_62212298_C_T | VPS13C | ARG-HIS | 0.056 | 0.004 | 2 |
| chr15_66621382_G_A | DIS3L | ARG-HIS | 0.053 | 0.004 | 2 |
| chr17_28651840_C_T | TMIGD1 | VAL-ILE | 0.053 | 0.004 | 2 |
| chr17_42849660_G_A | ADAM11 | ARG-GLN | 0.063 | 0.004 | 2 |
| chr19_15905414_A_C | OR10H5 | LYS-GLN | 0.053 | 0.004 | 2 |
| chr19_47280263_T_G | SLC1A5 | ILE-LEU | 0.053 | 0.004 | 2 |
| chr19_48602951_C_A | PLA2G4C | VAL-PHE | 0.053 | 0.004 | 2 |
| chrX_37663322_G_C | CYBB | GLY-ARG | 0.053 | 0.004 | 2 |
| chrX_102962435_T_C | GLRA4 | ASP-GLY | 0.053 | 0.004 | 2 |
| chr1_17588689_T_A | PADI3 | LEU-HIS | 0.083 | 0.005 | 3 |
| chr1_23418803_G_C | LUZP1 | SER-CYS | 0.079 | 0.005 | 3 |
| chr1_25291006_G_T | RUNX3 | none | 0.056 | 0.005 | 2 |
| chr1_26526375_G_A | CATSPER4 | none | 0.053 | 0.005 | 2 |
| chr1_57383357_C_T | C8A | PRO-SER | 0.053 | 0.005 | 2 |
| chr1_86961291_C_G | CLCA1 | ASN-LYS | 0.053 | 0.005 | 2 |
| chr1_92163646_G_A | TGFBR3 | PRO-SER | 0.056 | 0.005 | 2 |
| chr1_109837853_G_A | MYBPHL | HIS-TYR | 0.088 | 0.005 | 3 |
| chr1_205388384_C_G | LEMD1 | GLY-ALA | 0.053 | 0.005 | 2 |
| chr1_214551420_C_T | PTPN14 | GLY-ASP | 0.053 | 0.005 | 2 |
| chr2_55407644_A_T | C2orf63 | none | 0.056 | 0.005 | 2 |
| chr2_160604372_A_G | 7-Mar | MET-VAL | 0.053 | 0.005 | 2 |
| chr2_228388517_T_C | AGFG1 | VAL-ALA | 0.053 | 0.005 | 2 |
| chr3_27387641_C_T | NEK10 | GLY-SER | 0.125 | 0.005 | 2 |
| chr3_150398311_C_A | FAM194A | ARG-LEU | 0.053 | 0.005 | 2 |
| chr3_193185128_A_G | ATP13A4 | VAL-ALA | 0.053 | 0.005 | 2 |
| chr4_41686520_C_T | LIMCH1 | PRO-SER | 0.053 | 0.005 | 2 |
| chr4_55127448_G_A | PDGFRA | GLY-ASP | 0.053 | 0.005 | 2 |
| chr4_102751014_G_C | BANK1 | TRP-CYS | 0.094 | 0.005 | 2 |
| chr4_102951333_G_A | BANK1 | ARG-GLN | 0.053 | 0.005 | 2 |
| chr5_55272085_G_C | IL6ST | LEU-VAL | 0.053 | 0.005 | 2 |
| chr5_102338739_C_G | PAM | SER-TRP | 0.071 | 0.005 | 2 |
| chr5_110428060_T_C | WDR36 | LEU-PRO | 0.053 | 0.005 | 2 |
| chr5_180030322_C_T | FLT4 | ARG-GLN | 0.100 | 0.005 | 3 |
| chr6_51637536_G_T | PKHD1 | THR-LYS | 0.053 | 0.005 | 2 |
| chr6_110763935_T_C | SLC22A16 | GLU-GLY | 0.053 | 0.005 | 2 |
| chr6_111983038_G_C | FYN | ASP-GLU | 0.053 | 0.005 | 2 |
| chr8_8185746_T_C | SGK223 | HIS-ARG | 0.088 | 0.005 | 2 |
| chr8_22398155_A_G | PPP3CC | LYS-ARG | 0.053 | 0.005 | 2 |
| chr8_82357149_A_G | PMP2 | ILE-THR | 0.056 | 0.005 | 2 |
| chr9_80020874_C_A | VPS13A | HIS-ASN | 0.053 | 0.005 | 2 |
| chr9_107266833_G_A | OR13F1 | CYS-TYR | 0.053 | 0.005 | 2 |
| chr10_7765529_A_G | ITIH2 | GLN-ARG | 0.056 | 0.005 | 2 |
| chr10_46121886_C_T | ANUBL1 | ARG-GLN | 0.056 | 0.005 | 2 |
| chr10_51582911_C_T | NCOA4 | THR-ILE | 0.053 | 0.005 | 2 |
| chr10_70760271_T_C | KIAA1279 | MET-THR | 0.079 | 0.005 | 2 |
| chr10_135025208_A_G | KNDC1 | LYS-ARG | 0.059 | 0.005 | 2 |
| chr11_28106253_G_A | KIF18A | PRO-SER | 0.056 | 0.005 | 2 |
| chr11_130784886_T_C | SNX19 | SER-GLY | 0.053 | 0.005 | 2 |
| chr12_48143315_A_G | RAPGEF3 | LEU-PRO | 0.079 | 0.005 | 3 |
| chr12_48597051_T_G | OR10AD1 | THR-PRO | 0.053 | 0.005 | 2 |
| chr12_58020582_G_A | B4GALNT1 | ALA-VAL | 0.079 | 0.005 | 3 |
| chr12_124856830_C_T | NCOR2 | GLU-LYS | 0.079 | 0.005 | 2 |
| chr13_47345630_C_T | ESD | GLY-ASP | 0.056 | 0.005 | 2 |
| chr14_23282336_G_A | SLC7A7 | ALA-VAL | 0.053 | 0.005 | 2 |
| chr14_23451345_G_A | JUB | PRO-LEU | 0.053 | 0.005 | 2 |
| chr14_24572932_G_A | PCK2 | ARG-GLN | 0.053 | 0.005 | 2 |
| chr14_95906095_C_A | C14orf49 | LYS-ASN | 0.053 | 0.005 | 2 |
| chr16_2813096_C_G | SRRM2 | THR-ARG | 0.079 | 0.005 | 3 |
| chr16_15808876_C_G | MYH11,NDE1 | GLU-ASP | 0.053 | 0.005 | 2 |
| chr16_24950880_C_T | ARHGAP17 | ARG-GLN | 0.063 | 0.005 | 2 |
| chr16_53504536_A_T | RBL2 | ARG-SER | 0.053 | 0.005 | 2 |
| chr16_89350911_G_C | ANKRD11 | THR-SER | 0.053 | 0.005 | 2 |
| chr17_8053916_G_A | PER1 | PRO-SER | 0.053 | 0.005 | 2 |
| chr17_47784374_G_C | SLC35B1 | THR-SER | 0.053 | 0.005 | 2 |
| chr18_67833306_T_C | RTTN | ILE-VAL | 0.056 | 0.005 | 2 |
| chr19_17476933_G_T | PLVAP | PHE-LEU | 0.053 | 0.005 | 2 |
| chr19_38202515_G_A | ZNF607 | SER-PHE | 0.368 | 0.005 | 11 |
| chr19_48968015_C_T | KCNJ14 | ALA-VAL | 0.079 | 0.005 | 3 |
| chr19_55144710_A_T | LILRB1 | GLN-LEU | 0.053 | 0.005 | 2 |
| chr21_44190901_T_G | PDE9A | MET-ARG | 0.053 | 0.005 | 2 |
| chr22_25158445_G_A | PIWIL3 | ARG-CYS | 0.053 | 0.005 | 2 |
| chr22_37866063_G_A | MFNG | ARG-CYS | 0.056 | 0.005 | 2 |
| chrX_107844666_G_T | COL4A5 | LYS-ASN | 0.053 | 0.005 | 2 |
| chr1_115142870_G_A | DENND2C | SER-PHE | 0.053 | 0.006 | 2 |
| chr1_248487701_G_A | OR2M7 | THR-ILE | 0.053 | 0.006 | 2 |
| chr2_215617178_C_G | BARD1 | CYS-SER | 0.056 | 0.006 | 2 |
| chr2_219678877_C_T | CYP27A1 | PRO-LEU | 0.053 | 0.006 | 2 |
| chr3_46007825_T_C | FYCO1 | ASN-ASP | 0.079 | 0.006 | 3 |
| chr3_123451932_G_A | MYLK | PRO-SER | 0.059 | 0.006 | 2 |
| chr4_71024463_G_C | C4orf40 | GLY-ALA | 0.059 | 0.006 | 2 |
| chr5_1254594_C_T | TERT | ALA-THR | 0.079 | 0.006 | 3 |
| chr5_169028316_C_A | CCDC99 | ARG-SER | 0.053 | 0.006 | 2 |
| chr5_171777393_G_A | SH3PXD2B | PRO-LEU | 0.063 | 0.006 | 2 |
| chr7_92730745_C_T | SAMD9 | ALA-THR | 0.063 | 0.006 | 2 |
| chr7_127015083_G_A | ZNF800 | PRO-SER | 0.059 | 0.006 | 2 |
| chr8_17513484_T_C | MTUS1 | GLU-GLY | 0.053 | 0.006 | 2 |
| chr9_35042296_G_A | C9orf131 | MET-ILE | 0.053 | 0.006 | 2 |
| chr9_71998936_C_T | FAM189A2 | THR-ILE | 0.056 | 0.006 | 2 |
| chr10_75258901_C_G | USP54 | GLY-ALA | 0.053 | 0.006 | 2 |
| chr11_26587018_C_T | ANO3,MUC15 | ASP-ASN | 0.053 | 0.006 | 2 |
| chr11_35226083_C_T | CD44 | THR-MET | 0.053 | 0.006 | 2 |
| chr12_52885433_G_T | KRT6A | LEU-MET | 0.053 | 0.006 | 2 |
| chr12_101018061_C_T | GAS2L3 | SER-LEU | 0.053 | 0.006 | 2 |
| chr17_39521494_G_A | KRT33B | ALA-VAL | 0.053 | 0.006 | 2 |
| chr17_79639684_G_A | CCDC137 | GLY-ARG | 0.083 | 0.006 | 2 |
| chr19_53058429_C_T | ZNF808 | ARG-CYS | 0.053 | 0.006 | 2 |
| chr19_55144711_G_C | LILRB1 | GLN-HIS | 0.053 | 0.006 | 2 |
| chr20_33586968_G_A | MYH7B | ASP-ASN | 0.079 | 0.006 | 3 |
| chr22_40364122_G_A | GRAP2 | ARG-GLN | 0.125 | 0.006 | 2 |
| chrX_3248104_C_T | MXRA5 | ASP-ASN | 0.053 | 0.006 | 2 |
| chrX_38020288_G_A | SRPX | PRO-SER | 0.056 | 0.006 | 2 |
| chr1_8074334_C_T | ERRFI1 | ASP-ASN | 0.053 | 0.007 | 2 |
| chr1_17660468_C_A | PADI4 | PRO-THR | 0.053 | 0.007 | 2 |
| chr1_104162351_A_G | AMY2A | HIS-ARG | 0.053 | 0.007 | 2 |
| chr2_183616913_A_C | DNAJC10 | MET-LEU | 0.056 | 0.007 | 2 |
| chr3_169710614_G_C | SEC62 | GLU-ASP | 0.053 | 0.007 | 2 |
| chr4_1165131_C_T | SPON2 | ALA-THR | 0.107 | 0.007 | 2 |
| chr4_8235220_C_A | SH3TC1 | LEU-MET | 0.105 | 0.007 | 4 |
| chr4_47954624_C_T | CNGA1 | ARG-GLN | 0.056 | 0.007 | 2 |
| chr4_57797350_C_G | REST | PRO-ALA | 0.079 | 0.007 | 3 |
| chr4_139144395_T_C | SLC7A11 | ILE-VAL | 0.053 | 0.007 | 2 |
| chr5_32074462_G_A | PDZD2 | ALA-THR | 0.079 | 0.007 | 3 |
| chr5_44809446_g_C | MRPS30 | GLU-GLN | 0.077 | 0.007 | 2 |
| chr5_159686752_C_T | CCNJL | VAL-ILE | 0.053 | 0.007 | 2 |
| chr5_176005481_G_A | CDHR2 | GLY-ARG | 0.079 | 0.007 | 3 |
| chr6_29054914_T_A | OR2B3 | THR-SER | 0.079 | 0.007 | 3 |
| chr6_38820493_C_A | DNAH8 | ASP-GLU | 0.053 | 0.007 | 2 |
| chr6_41903798_C_A | CCND3 | GLU-ASP | 0.059 | 0.007 | 2 |
| chr6_90660954_G_C | BACH2 | LEU-VAL | 0.053 | 0.007 | 2 |
| chr6_111583530_G_C | KIAA1919 | SER-THR | 0.053 | 0.007 | 2 |
| chr6_160953642_A_G | LPA | LEU-PRO | 0.053 | 0.007 | 2 |
| chr7_4002309_C_G | SDK1 | GLN-GLU | 0.053 | 0.007 | 2 |
| chr7_7530225_C_A | COL28A1 | GLY-VAL | 0.059 | 0.007 | 2 |
| chr7_12263971_G_A | TMEM106B | SER-ASN | 0.056 | 0.007 | 2 |
| chr7_151810476_A_G | GALNT11 | GLU-GLY | 0.053 | 0.007 | 2 |
| chr9_104335619_T_C | GRIN3A | ASN-SER | 0.053 | 0.007 | 2 |
| chr9_125391012_G_C | OR1B1 | PRO-ARG | 0.053 | 0.007 | 2 |
| chr10_105762909_C_G | SLK | ALA-GLY | 0.053 | 0.007 | 2 |
| chr10_127693479_A_G | FANK1 | HIS-ARG | 0.053 | 0.007 | 2 |
| chr11_124016058_C_T | VWA5A | ARG-CYS | 0.053 | 0.007 | 2 |
| chr12_40740686_A_G | LRRK2 | ASN-ASP | 0.100 | 0.007 | 3 |
| chr12_56532009_C_T | ESYT1 | ARG-CYS | 0.053 | 0.007 | 2 |
| chr13_41515118_C_T | ELF1 | GLU-LYS | 0.053 | 0.007 | 2 |
| chr13_94197611_C_T | GPC6 | LEU-PHE | 0.053 | 0.007 | 2 |
| chr14_24683304_C_A | MDP1,NEDD8-MDP1 | ARG-MET | 0.053 | 0.007 | 2 |
| chr14_30046511_T_C | PRKD1 | HIS-ARG | 0.053 | 0.007 | 2 |
| chr14_68352672_A_G | RAD51B | TYR-CYS | 0.053 | 0.007 | 2 |
| chr14_73421159_A_G | DCAF4 | SER-GLY | 0.053 | 0.007 | 2 |
| chr14_94844947_C_T | SERPINA1 | GLU-LYS | 0.053 | 0.007 | 2 |
| chr16_2296877_C_T | ECI1 | GLY-SER | 0.053 | 0.007 | 2 |
| chr16_29891206_C_T | SEZ6L2 | ASP-ASN | 0.079 | 0.007 | 3 |
| chr16_72170408_C_A | PMFBP1 | ARG-LEU | 0.053 | 0.007 | 2 |
| chr17_6012979_C_T | WSCD1 | THR-ILE | 0.053 | 0.007 | 2 |
| chr17_7669761_G_A | DNAH2 | GLU-LYS | 0.053 | 0.007 | 2 |
| chr17_14248376_A_G | HS3ST3B1 | ILE-VAL | 0.083 | 0.007 | 3 |
| chr17_37815304_G_C | STARD3 | GLY-ALA | 0.053 | 0.007 | 2 |
| chr17_38450248_G_A | CDC6 | ASP-ASN | 0.053 | 0.007 | 2 |
| chr17_39521142_C_T | KRT33B | ARG-HIS | 0.079 | 0.007 | 3 |
| chr17_66366659_C_G | ARSG | ARG-GLY | 0.053 | 0.007 | 2 |
| chr17_79205672_G_A | C17orf56 | ARG-TRP | 0.053 | 0.007 | 2 |
| chr19_1008645_C_A | GRIN3B | ALA-GLU | 0.053 | 0.007 | 2 |
| chr19_7708058_C_T | STXBP2 | THR-MET | 0.053 | 0.007 | 2 |
| chr19_10132318_T_C | RDH8 | TYR-HIS | 0.053 | 0.007 | 2 |
| chr19_21132072_T_C | ZNF85 | ILE-THR | 0.056 | 0.007 | 2 |
| chr19_32845600_G_A | ZNF507 | ASP-ASN | 0.053 | 0.007 | 2 |
| chr19_38652962_C_T | SIPA1L3 | PRO-LEU | 0.053 | 0.007 | 2 |
| chr19_54744919_T_A | LILRA6 | ASP-VAL | 0.079 | 0.007 | 3 |
| chr19_58452599_T_G | ZNF256 | LYS-THR | 0.053 | 0.007 | 2 |
| chrX_107315933_G_A | VSIG1 | VAL-ILE | 0.053 | 0.007 | 2 |
| chr1_1959699_G_A | GABRD | ARG-HIS | 0.059 | 0.008 | 2 |
| chr1_154072601_G_A | NUP210L | PRO-LEU | 0.056 | 0.008 | 2 |
| chr2_141773450_T_C | LRP1B | ILE-VAL | 0.053 | 0.008 | 2 |
| chr3_44488293_T_C | ZNF445 | GLU-GLY | 0.053 | 0.008 | 2 |
| chr3_167512569_G_A | SERPINI1 | ALA-THR | 0.079 | 0.008 | 3 |
| chr5_36219710_C_T | NADKD1 | ARG-HIS | 0.056 | 0.008 | 2 |
| chr5_139905844_G_A | ANKHD1-EIF4EBP3 | GLY-SER | 0.063 | 0.008 | 2 |
| chr5_140530718_G_T | PCDHB6 | ALA-SER | 0.053 | 0.008 | 2 |
| chr6_136882717_G_A | MAP3K5 | THR-ILE | 0.053 | 0.008 | 2 |
| chr7_92085763_C_T | GATAD1 | ARG-TRP | 0.053 | 0.008 | 2 |
| chr8_94747496_A_C | RBM12B | ASP-GLU | 0.056 | 0.008 | 2 |
| chr9_103947810_T_G | LPPR1 | SER-ALA | 0.079 | 0.008 | 3 |
| chr9_136913527_G_A | BRD3 | ALA-VAL | 0.059 | 0.008 | 2 |
| chr10_95347041_G_A | O3FAR1 | ARG-HIS | 0.053 | 0.008 | 2 |
| chr10_97096357_C_T | SORBS1 | GLY-GLU | 0.088 | 0.008 | 3 |
| chr11_89896537_A_G | NAALAD2 | THR-ALA | 0.053 | 0.008 | 2 |
| chr11_102401411_A_C | MMP7 | CYS-TRP | 0.056 | 0.008 | 2 |
| chr14_20444393_T_C | OR4K15 | VAL-ALA | 0.053 | 0.008 | 2 |
| chr15_40914831_C_G | CASC5 | THR-SER | 0.056 | 0.008 | 2 |
| chr16_3293880_A_G | MEFV | ILE-THR | 0.053 | 0.008 | 2 |
| chr16_4733253_C_T | MGRN1 | SER-LEU | 0.079 | 0.008 | 3 |
| chr16_49669923_G_A | ZNF423 | ALA-VAL | 0.053 | 0.008 | 2 |
| chr17_5338281_G_A | C1QBP | THR-MET | 0.105 | 0.008 | 4 |
| chr17_74019453_G_A | EVPL | ARG-CYS | 0.053 | 0.008 | 2 |
| chr17_77079575_G_A | ENGASE | ARG-GLN | 0.053 | 0.008 | 2 |
| chr17_77082174_C_T | ENGASE | ARG-CYS | 0.053 | 0.008 | 2 |
| chr17_78184601_C_T | SGSH | VAL-MET | 0.053 | 0.008 | 2 |
| chr21_15873026_T_C | SAMSN1 | THR-ALA | 0.053 | 0.008 | 2 |
| chrX_102004317_A_G | BHLHB9 | SER-GLY | 0.053 | 0.008 | 2 |
| chr1_16332665_C_T | C1orf64 | LEU-PHE | 0.088 | 0.009 | 2 |
| chr1_51868160_C_G | EPS15 | GLY-ALA | 0.053 | 0.009 | 2 |
| chr1_82416040_C_T | LPHN2 | PRO-SER | 0.053 | 0.009 | 2 |
| chr1_159890163_T_A | TAGLN2 | GLN-LEU | 0.079 | 0.009 | 2 |
| chr1_220161969_T_C | EPRS | GLU-GLY | 0.056 | 0.009 | 2 |
| chr2_108910751_G_A | SULT1C2 | ARG-GLN | 0.053 | 0.009 | 2 |
| chr2_160604541_G_A | 7-Mar | SER-ASN | 0.088 | 0.009 | 3 |
| chr2_182542998_G_T | NEUROD1 | PRO-HIS | 0.053 | 0.009 | 2 |
| chr3_19575232_C_T | KCNH8 | PRO-SER | 0.053 | 0.009 | 2 |
| chr3_46712490_G_A | ALS2CL | SER-PHE | 0.105 | 0.009 | 4 |
| chr4_95376504_A_G | PDLIM5 | LYS-ARG | 0.053 | 0.009 | 2 |
| chr5_38921864_G_A | OSMR | GLY-ASP | 0.053 | 0.009 | 2 |
| chr5_133901939_G_T | PHF15 | GLY-VAL | 0.056 | 0.009 | 2 |
| chr6_30862440_A_G | DDR1 | ASN-SER | 0.079 | 0.009 | 3 |
| chr6_97599674_G_A | MMS22L | PRO-LEU | 0.053 | 0.009 | 2 |
| chr6_152784621_T_C | SYNE1 | GLN-ARG | 0.056 | 0.009 | 2 |
| chr7_1479642_C_A | MICALL2 | ALA-SER | 0.056 | 0.009 | 2 |
| chr7_18067261_G_C | PRPS1L1 | ARG-GLY | 0.079 | 0.009 | 3 |
| chr7_87537188_A_G | DBF4 | LYS-GLU | 0.063 | 0.009 | 2 |
| chr7_89856433_G_A | STEAP2 | GLY-GLU | 0.053 | 0.009 | 2 |
| chr8_100205255_G_A | VPS13B | ALA-THR | 0.056 | 0.009 | 2 |
| chr9_140400464_C_T | PNPLA7 | GLU-LYS | 0.088 | 0.009 | 2 |
| chr11_4903500_G_A | OR51T1 | ARG-HIS | 0.079 | 0.009 | 3 |
| chr11_77090939_C_T | PAK1 | none | 0.056 | 0.009 | 2 |
| chr12_46320944_C_T | SCAF11 | ARG-HIS | 0.053 | 0.009 | 2 |
| chr12_52285090_G_A | ANKRD33 | GLY-GLU | 0.053 | 0.009 | 2 |
| chr12_56630444_G_C | SLC39A5 | SER-THR | 0.056 | 0.009 | 2 |
| chr12_57539082_C_T | LRP1 | ALA-VAL | 0.053 | 0.009 | 2 |
| chr12_106729425_G_A | TCP11L2 | ASP-ASN | 0.063 | 0.009 | 2 |
| chr14_39818076_A_G | CTAGE5 | ARG-GLY | 0.053 | 0.009 | 2 |
| chr14_73733285_G_A | PAPLN | ALA-THR | 0.053 | 0.009 | 2 |
| chr16_12145791_G_C | SNX29 | GLY-ALA | 0.053 | 0.009 | 2 |
| chr19_8182174_G_A | FBN3 | none | 0.053 | 0.009 | 2 |
| chr19_15350625_C_T | BRD4 | ARG-HIS | 0.056 | 0.009 | 2 |
| chr19_41383849_C_G | CYP2A7 | SER-THR | 0.053 | 0.009 | 2 |
| chr20_61391378_A_G | NTSR1 | TYR-CYS | 0.053 | 0.009 | 2 |
| chr1_14105049_C_A | PRDM2 | ASP-GLU | 0.053 | 0.010 | 2 |
| chr1_14107135_C_G | PRDM2 | PRO-ALA | 0.053 | 0.010 | 2 |
| chr1_109810200_G_A | CELSR2 | ARG-LYS | 0.079 | 0.010 | 3 |
| chr1_145583938_C_G | PIAS3 | SER-CYS | 0.053 | 0.010 | 2 |
| chr1_155206167_C_T | GBA | GLU-LYS | 0.053 | 0.010 | 2 |
| chr1_247875313_G_A | OR6F1 | LEU-PHE | 0.053 | 0.010 | 2 |
| chr2_17947886_G_A | GEN1 | SER-ASN | 0.053 | 0.010 | 2 |
| chr2_27438615_G_C | C2orf28 | ASP-HIS | 0.053 | 0.010 | 2 |
| chr2_160690656_G_A | LY75,LY75-CD302 | PRO-LEU | 0.083 | 0.010 | 3 |
| chr2_220251685_T_G | DNPEP | ASN-HIS | 0.079 | 0.010 | 3 |
| chr3_32533246_T_C | CMTM6 | THR-ALA | 0.111 | 0.010 | 4 |
| chr3_150384657_G_A | FAM194A | ARG-CYS | 0.079 | 0.010 | 3 |
| chr4_100503136_C_G | MTTP | ARG-GLY | 0.053 | 0.010 | 2 |
| chr4_106395144_T_C | PPA2 | THR-ALA | 0.083 | 0.010 | 2 |
| chr5_23527559_A_G | PRDM9 | LYS-GLU | 0.053 | 0.010 | 2 |
| chr5_34911914_G_C | RAD1 | THR-SER | 0.079 | 0.010 | 3 |
| chr5_149360630_C_T | SLC26A2 | ARG-TRP | 0.118 | 0.010 | 3 |
| chr5_150947162_A_G | FAT2 | VAL-ALA | 0.079 | 0.010 | 3 |
| chr5_151771874_C_T | NMUR2 | GLU-LYS | 0.079 | 0.010 | 3 |
| chr5_176409574_G_A | UIMC1 | ARG-TRP | 0.056 | 0.010 | 2 |
| chr6_20109956_A_G | MBOAT1 | PHE-LEU | 0.053 | 0.010 | 2 |
| chr6_45916999_G_T | CLIC5 | PRO-HIS | 0.079 | 0.010 | 3 |
| chr6_106553096_G_A | PRDM1 | SER-ASN | 0.053 | 0.010 | 2 |
| chr6_142400020_G_T | NMBR | PRO-HIS | 0.053 | 0.010 | 2 |
| chr7_100465824_G_A | TRIP6 | ARG-GLN | 0.088 | 0.010 | 3 |
| chr7_127965924_T_G | RBM28 | MET-LEU | 0.053 | 0.010 | 2 |
| chr8_41582031_G_T | ANK1 | ASN-LYS | 0.056 | 0.010 | 2 |
| chr9_138439086_T_A | OBP2A | PHE-TYR | 0.053 | 0.010 | 2 |
| chr12_52962167_T_C | KRT74 | SER-GLY | 0.056 | 0.010 | 2 |
| chr12_132624434_T_C | DDX51 | GLN-ARG | 0.059 | 0.010 | 2 |
| chr13_52971517_C_T | THSD1 | GLU-LYS | 0.079 | 0.010 | 3 |
| chr14_23564437_T_C | ACIN1 | ASN-SER | 0.079 | 0.010 | 2 |
| chr14_24040430_C_T | JPH4 | ALA-THR | 0.053 | 0.010 | 2 |
| chr14_69341658_G_A | ACTN1 | PRO-LEU | 0.056 | 0.010 | 2 |
| chr14_73961982_C_T | HEATR4 | GLY-GLU | 0.079 | 0.010 | 3 |
| chr14_74036500_C_T | ACOT2 | ARG-TRP | 0.125 | 0.010 | 2 |
| chr15_42434824_C_T | PLA2G4F | ARG-HIS | 0.079 | 0.010 | 3 |
| chr15_43317071_T_C | UBR1 | ILE-VAL | 0.111 | 0.010 | 4 |
| chr16_57283730_G_A | ARL2BP | GLU-LYS | 0.053 | 0.010 | 2 |
| chr16_67434917_C_T | ZDHHC1 | ARG-GLN | 0.079 | 0.010 | 3 |
| chr16_69143816_G_A | HAS3 | ARG-HIS | 0.079 | 0.010 | 3 |
| chr17_33769039_G_A | SLFN13 | ARG-CYS | 0.053 | 0.010 | 2 |
| chr17_74018597_T_C | EVPL | TYR-CYS | 0.071 | 0.010 | 2 |
| chr17_76973249_C_T | LGALS3BP | VAL-MET | 0.053 | 0.010 | 2 |
| chr19_41383115_G_A | CYP2A7 | ARG-TRP | 0.079 | 0.010 | 3 |
| chr19_51582895_G_A | KLK14 | ARG-CYS | 0.077 | 0.010 | 2 |
| chr19_52934741_A_C | ZNF534 | GLN-PRO | 0.056 | 0.010 | 2 |
| chr20_7864284_T_C | HAO1 | LYS-GLU | 0.056 | 0.010 | 2 |
| chr20_31607551_G_C | BPIFB2 | ASP-HIS | 0.056 | 0.010 | 2 |
| chr20_62172244_C_G | SRMS | SER-THR | 0.088 | 0.010 | 3 |
| chrX_46719498_C_T | RP2 | ARG-TRP | 0.056 | 0.010 | 2 |
| chrX_103267974_G_A | H2BFWT | ARG-TRP | 0.067 | 0.010 | 2 |
| chr1_19203725_A_G | ALDH4A1 | PHE-LEU | 0.111 | 0.011 | 3 |
| chr1_67833643_G_A | IL12RB2 | GLY-ASP | 0.056 | 0.011 | 2 |
| chr1_155175089_C_T | THBS3 | ARG-GLN | 0.053 | 0.011 | 2 |
| chr1_207868022_G_T | CR1L | GLY-VAL | 0.143 | 0.011 | 2 |
| chr1_212273606_G_A | DTL | SER-ASN | 0.053 | 0.011 | 2 |
| chr2_24435599_G_A | ITSN2 | ARG-TRP | 0.079 | 0.011 | 3 |
| chr2_120413984_C_G | PCDP1 | LEU-VAL | 0.053 | 0.011 | 2 |
| chr4_57181629_C_T | KIAA1211 | PRO-LEU | 0.250 | 0.011 | 4 |
| chr4_162697058_C_T | FSTL5 | GLY-GLU | 0.059 | 0.011 | 2 |
| chr5_121786959_G_A | SNCAIP | ARG-HIS | 0.079 | 0.011 | 3 |
| chr6_36839598_C_G | PPIL1 | CYS-SER | 0.053 | 0.011 | 2 |
| chr6_155153307_G_A | SCAF8 | SER-ASN | 0.083 | 0.011 | 2 |
| chr7_84727240_A_G | SEMA3D | SER-PRO | 0.053 | 0.011 | 2 |
| chr8_6794366_G_T | DEFA4 | ALA-GLU | 0.053 | 0.011 | 2 |
| chr10_95161268_C_T | MYOF | ASP-ASN | 0.053 | 0.011 | 2 |
| chr10_101667814_A_G | DNMBP | MET-THR | 0.056 | 0.011 | 2 |
| chr10_113926224_A_G | GPAM | ILE-THR | 0.053 | 0.011 | 2 |
| chr11_376331_T_A | B4GALNT4 | PHE-TYR | 0.079 | 0.011 | 3 |
| chr11_7507174_C_T | OLFML1 | PRO-LEU | 0.079 | 0.011 | 3 |
| chr11_102584135_G_A | MMP8 | GLN-stop | 0.056 | 0.011 | 2 |
| chr12_51208122_C_G | ATF1 | PRO-ALA | 0.079 | 0.011 | 3 |
| chr13_24465537_C_T | C1QTNF9B-AS1 | GLY-GLU | 0.079 | 0.011 | 2 |
| chr15_65158047_G_A | PLEKHO2 | ARG-GLN | 0.053 | 0.011 | 2 |
| chr16_2003016_G_A | RPL3L | ALA-VAL | 0.053 | 0.011 | 2 |
| chr17_78166326_G_A | CARD14 | GLU-LYS | 0.059 | 0.011 | 2 |
| chr18_61323259_A_T | SERPINB3 | TRP-ARG | 0.056 | 0.011 | 2 |
| chr19_58190130_G_A | ZSCAN4 | GLU-LYS | 0.053 | 0.011 | 2 |
| chr20_31805464_C_A | BPIFA3 | ALA-GLU | 0.053 | 0.011 | 2 |
| chr1_17668509_G_A | PADI4 | GLY-SER | 0.079 | 0.012 | 3 |
| chr1_36937878_C_T | CSF3R | ASP-ASN | 0.053 | 0.012 | 2 |
| chr1_52854961_G_A | ORC1 | ALA-VAL | 0.105 | 0.012 | 4 |
| chr1_156146546_C_T | SEMA4A | PRO-SER | 0.053 | 0.012 | 2 |
| chr1_161130701_C_T | USP21 | PRO-SER | 0.105 | 0.012 | 4 |
| chr1_169484767_A_G | F5 | MET-THR | 0.083 | 0.012 | 2 |
| chr1_180886140_C_T | KIAA1614 | ARG-CYS | 0.056 | 0.012 | 2 |
| chr1_196918741_G_A | CFHR2 | CYS-TYR | 0.056 | 0.012 | 2 |
| chr1_246805265_A_G | CNST | GLN-ARG | 0.063 | 0.012 | 2 |
| chr1_248084440_T_G | OR2T8 | SER-ALA | 0.105 | 0.012 | 4 |
| chr2_165947079_C_A | SCN3A | GLY-CYS | 0.053 | 0.012 | 2 |
| chr2_185801103_A_C | ZNF804A | ASN-THR | 0.063 | 0.012 | 2 |
| chr3_50513613_C_T | CACNA2D2 | ARG-GLN | 0.067 | 0.012 | 2 |
| chr3_182583338_T_A | ATP11B | ILE-ASN | 0.079 | 0.012 | 3 |
| chr4_8588986_G_A | GPR78 | ALA-THR | 0.083 | 0.012 | 3 |
| chr4_107157623_G_A | TBCK | THR-MET | 0.079 | 0.012 | 3 |
| chr5_141335594_A_G | PCDH12 | LEU-PRO | 0.053 | 0.012 | 2 |
| chr6_31938120_C_T | DOM3Z | none | 0.053 | 0.012 | 2 |
| chr6_51497503_C_A | PKHD1 | ARG-LEU | 0.053 | 0.012 | 2 |
| chr6_56480500_T_C | DST | ILE-VAL | 0.083 | 0.012 | 3 |
| chr6_56485023_T_C | DST | LYS-ARG | 0.056 | 0.012 | 2 |
| chr7_99091172_g_A | ZNF394 | ARG-CYS | 0.053 | 0.012 | 2 |
| chr7_105148593_G_A | PUS7 | HIS-TYR | 0.079 | 0.012 | 3 |
| chr8_18080001_G_A | NAT1 | VAL-ILE | 0.053 | 0.012 | 2 |
| chr8_18080196_T_G | NAT1 | SER-ALA | 0.056 | 0.012 | 2 |
| chr9_15564100_C_T | C9orf93 | THR-ILE | 0.059 | 0.012 | 2 |
| chr10_16932490_G_T | CUBN | LEU-ILE | 0.053 | 0.012 | 2 |
| chr10_51465429_C_T | AGAP7 | ASP-ASN | 0.053 | 0.012 | 2 |
| chr11_3690558_G_T | CHRNA10 | THR-ASN | 0.053 | 0.012 | 2 |
| chr11_15243047_G_A | INSC | ASP-ASN | 0.083 | 0.012 | 2 |
| chr12_38714242_G_A | ALG10B | GLU-LYS | 0.079 | 0.012 | 3 |
| chr12_52758810_C_T | KRT85 | ASP-ASN | 0.053 | 0.012 | 2 |
| chr13_24233219_C_T | TNFRSF19 | ALA-VAL | 0.053 | 0.012 | 2 |
| chr14_20874411_T_C | TEP1 | HIS-ARG | 0.053 | 0.012 | 2 |
| chr14_51372238_T_A | PYGL | ILE-LEU | 0.053 | 0.012 | 2 |
| chr14_58605072_C_G | C14orf37 | GLU-ASP | 0.053 | 0.012 | 2 |
| chr14_74759059_G_C | ABCD4 | THR-ARG | 0.053 | 0.012 | 2 |
| chr14_78161131_C_T | ALKBH1 | MET-ILE | 0.053 | 0.012 | 2 |
| chr15_31221493_C_T | FAN1 | PRO-SER | 0.053 | 0.012 | 2 |
| chr16_16276345_C_T | ABCC6 | ARG-LYS | 0.053 | 0.012 | 2 |
| chr17_8222870_C_T | ARHGEF15 | ARG-TRP | 0.056 | 0.012 | 2 |
| chr17_11881356_G_A | ZNF18 | SER-LEU | 0.079 | 0.012 | 3 |
| chr18_2890796_G_A | EMILIN2 | ARG-GLN | 0.105 | 0.012 | 4 |
| chr19_35758745_G_C | LSR | GLU-GLN | 0.053 | 0.012 | 2 |
| chr19_38573347_T_C | SIPA1L3 | MET-THR | 0.067 | 0.012 | 2 |
| chr19_51563265_G_A | KLK13 | HIS-TYR | 0.053 | 0.012 | 2 |
| chr20_54945309_T_C | AURKA | MET-VAL | 0.053 | 0.012 | 2 |
| chr21_45542072_T_A | PWP2 | PHE-ILE | 0.053 | 0.012 | 2 |
| chr22_45996298_A_G | FBLN1 | HIS-ARG | 0.083 | 0.012 | 3 |
| chr1_1334475_G_C | CCNL2 | THR-SER | 0.083 | 0.013 | 2 |
| chr1_17662705_G_C | PADI4 | ARG-THR | 0.053 | 0.013 | 2 |
| chr1_40703244_G_A | RLF | GLY-ASP | 0.079 | 0.013 | 3 |
| chr1_110302450_C_T | EPS8L3 | MET-ILE | 0.053 | 0.013 | 2 |
| chr1_117753455_A_G | VTCN1 | LEU-PRO | 0.105 | 0.013 | 4 |
| chr1_196967354_G_A | CFHR5 | ARG-HIS | 0.067 | 0.013 | 2 |
| chr1_248263471_G_A | OR2L13 | ARG-HIS | 0.053 | 0.013 | 2 |
| chr1_248308934_C_T | OR2M5 | ALA-VAL | 0.053 | 0.013 | 2 |
| chr2_27308159_G_A | EMILIN1 | GLU-LYS | 0.053 | 0.013 | 2 |
| chr3_49760431_G_C | GMPPB | HIS-ASP | 0.053 | 0.013 | 2 |
| chr3_187447701_C_A | BCL6 | GLU-ASP | 0.053 | 0.013 | 2 |
| chr3_197403844_T_C | KIAA0226 | ASN-SER | 0.053 | 0.013 | 2 |
| chr4_113352075_A_G | ALPK1 | THR-ALA | 0.056 | 0.013 | 2 |
| chr4_113352832_C_T | ALPK1 | SER-PHE | 0.053 | 0.013 | 2 |
| chr4_126412154_G_A | FAT4 | ARG-LYS | 0.079 | 0.013 | 3 |
| chr5_154307070_G_C | GEMIN5 | ARG-GLY | 0.056 | 0.013 | 2 |
| chr5_176825069_C_T | SLC34A1 | HIS-TYR | 0.053 | 0.013 | 2 |
| chr6_10964015_G_A | SYCP2L | none | 0.053 | 0.013 | 2 |
| chr6_30610758_G_T | ATAT1 | GLY-VAL | 0.059 | 0.013 | 2 |
| chr6_108197775_G_A | SEC63 | THR-ILE | 0.079 | 0.013 | 2 |
| chr6_168297647_A_G | MLLT4 | ILE-VAL | 0.083 | 0.013 | 2 |
| chr7_130023561_G_A | CPA1 | ALA-THR | 0.053 | 0.013 | 2 |
| chr10_134996954_G_A | KNDC1 | ARG-GLN | 0.077 | 0.013 | 2 |
| chr11_397063_C_A | PKP3 | PRO-THR | 0.063 | 0.013 | 2 |
| chr11_6816792_T_C | OR6A2 | ILE-VAL | 0.053 | 0.013 | 2 |
| chr11_86158235_C_T | ME3 | ALA-THR | 0.053 | 0.013 | 2 |
| chr12_49087325_T_C | CCNT1 | THR-ALA | 0.056 | 0.013 | 2 |
| chr12_49994785_G_A | FAM186B | THR-MET | 0.105 | 0.013 | 4 |
| chr12_108985976_G_A | TMEM119 | LEU-PHE | 0.059 | 0.013 | 2 |
| chr13_95953517_G_T | ABCC4 | LEU-ILE | 0.056 | 0.013 | 2 |
| chr13_103343308_T_C | METTL21C | ASN-SER | 0.053 | 0.013 | 2 |
| chr15_31334362_C_T | TRPM1 | VAL-MET | 0.053 | 0.013 | 2 |
| chr16_81232336_T_C | PKD1L2 | THR-ALA | 0.059 | 0.013 | 2 |
| chr17_663472_C_A | GLOD4 | ALA-SER | 0.105 | 0.013 | 4 |
| chr17_2227655_G_T | TSR1,SRR | HIS-GLN | 0.056 | 0.013 | 2 |
| chr17_33679943_C_T | SLFN11 | SER-ASN | 0.053 | 0.013 | 2 |
| chr17_73839326_C_T | UNC13D | ALA-THR | 0.056 | 0.013 | 2 |
| chr18_51880889_G_A | STARD6 | ARG-stop | 0.083 | 0.013 | 2 |
| chr19_42231159_G_A | CEACAM5 | GLY-ARG | 0.053 | 0.013 | 2 |
| chr19_53611802_T_G | ZNF415 | ASN-THR | 0.056 | 0.013 | 2 |
| chr19_55086249_T_C | LILRA2 | LEU-SER | 0.053 | 0.013 | 2 |
| chr19_58118418_C_T | ZNF530 | HIS-TYR | 0.053 | 0.013 | 2 |
| chr21_34997018_C_T | CRYZL1 | ALA-THR | 0.056 | 0.013 | 2 |
| chrX_18972497_C_G | PHKA2 | GLU-GLN | 0.056 | 0.013 | 2 |
| chr1_117568217_G_A | CD101 | VAL-ILE | 0.053 | 0.014 | 2 |
| chr1_152277554_G_A | FLG | ARG-CYS | 0.079 | 0.014 | 3 |
| chr2_167141109_G_T | SCN9A | PRO-THR | 0.100 | 0.014 | 3 |
| chr2_210905194_T_C | C2orf67 | ASN-SER | 0.071 | 0.014 | 2 |
| chr3_197432000_C_T | KIAA0226 | ARG-GLN | 0.053 | 0.014 | 2 |
| chr4_2172456_T_C | POLN | SER-GLY | 0.053 | 0.014 | 2 |
| chr5_86564564_c_T | RASA1 | ALA-VAL | 0.079 | 0.014 | 3 |
| chr5_127873094_G_A | FBN2 | ALA-VAL | 0.107 | 0.014 | 3 |
| chr9_21970916_C_T | CDKN2A | ALA-THR | 0.079 | 0.014 | 3 |
| chr9_131107634_G_A | SLC27A4 | ARG-GLN | 0.059 | 0.014 | 2 |
| chr9_138439792_G_A | OBP2A | ARG-HIS | 0.088 | 0.014 | 3 |
| chr10_23295903_T_C | ARMC3 | SER-PRO | 0.053 | 0.014 | 2 |
| chr10_127668854_G_C | FANK1 | ARG-SER | 0.053 | 0.014 | 2 |
| chr10_134996950_A_G | KNDC1 | SER-GLY | 0.067 | 0.014 | 2 |
| chr11_55111500_T_C | OR4A16 | ILE-THR | 0.059 | 0.014 | 2 |
| chr11_86118683_C_A | CCDC81 | HIS-GLN | 0.083 | 0.014 | 3 |
| chr11_86126337_T_G | CCDC81 | LEU-ARG | 0.079 | 0.014 | 3 |
| chr12_26834858_G_A | ITPR2 | ALA-VAL | 0.079 | 0.014 | 3 |
| chr12_48358203_A_G | TMEM106C | THR-ALA | 0.083 | 0.014 | 3 |
| chr12_82752037_T_G | CCDC59 | ASN-THR | 0.053 | 0.014 | 2 |
| chr13_73319236_C_T | BORA | SER-LEU | 0.056 | 0.014 | 2 |
| chr13_96511868_T_A | UGGT2 | ASN-TYR | 0.067 | 0.014 | 2 |
| chr14_68159269_C_T | RDH11 | GLU-LYS | 0.053 | 0.014 | 2 |
| chr15_34159941_T_G | AVEN | GLU-ALA | 0.053 | 0.014 | 2 |
| chr15_41688732_C_T | NDUFAF1 | GLU-LYS | 0.053 | 0.014 | 2 |
| chr18_9944924_a_G | VAPA | ILE-VAL | 0.053 | 0.014 | 2 |
| chr19_21476107_T_C | ZNF708 | HIS-ARG | 0.056 | 0.014 | 2 |
| chr19_41847860_G_A | TGFB1 | THR-ILE | 0.079 | 0.014 | 3 |
| chr19_55998317_C_A | NAT14 | ASP-GLU | 0.136 | 0.014 | 3 |
| chr20_60902965_G_A | LAMA5 | ALA-VAL | 0.091 | 0.014 | 2 |
| chr22_50318061_G_C | CRELD2 | GLU-GLN | 0.059 | 0.014 | 2 |
| chrX_65418789_G_A | HEPH | ALA-THR | 0.053 | 0.014 | 2 |
| chr1_19166829_A_G | TAS1R2 | ILE-THR | 0.056 | 0.015 | 2 |
| chr1_94512565_C_T | ABCA4 | ARG-GLN | 0.079 | 0.015 | 3 |
| chr1_152282910_G_C | FLG | ASP-GLU | 0.079 | 0.015 | 3 |
| chr2_31593265_T_C | XDH | ILE-VAL | 0.132 | 0.015 | 5 |
| chr3_155200133_T_C | PLCH1 | MET-VAL | 0.053 | 0.015 | 2 |
| chr3_184070588_C_G | CLCN2 | GLU-ASP | 0.059 | 0.015 | 2 |
| chr3_186461524_C_T | KNG1 | ARG-stop | 0.079 | 0.015 | 3 |
| chr4_107847043_C_G | DKK2 | GLY-ARG | 0.053 | 0.015 | 2 |
| chr4_178363496_C_A | AGA | VAL-LEU | 0.053 | 0.015 | 2 |
| chr5_38406251_C_T | EGFLAM | ARG-CYS | 0.053 | 0.015 | 2 |
| chr5_176004680_A_C | CDHR2 | MET-LEU | 0.079 | 0.015 | 3 |
| chr5_176316455_C_T | HK3 | GLY-ARG | 0.056 | 0.015 | 2 |
| chr6_7565727_A_T | DSP | ILE-PHE | 0.079 | 0.015 | 3 |
| chr7_99454482_G_A | CYP3A43 | MET-ILE | 0.083 | 0.015 | 3 |
| chr7_104752798_G_A | MLL5 | SER-ASN | 0.053 | 0.015 | 2 |
| chr8_18730030_C_A | PSD3 | ARG-ILE | 0.053 | 0.015 | 2 |
| chr9_20988423_G_A | KIAA1797 | ASP-ASN | 0.056 | 0.015 | 2 |
| chr10_74881951_G_A | NUDT13 | GLY-ASP | 0.079 | 0.015 | 3 |
| chr10_99003811_G_A | ARHGAP19 | HIS-TYR | 0.053 | 0.015 | 2 |
| chr10_127705856_T_C | ADAM12 | ILE-VAL | 0.056 | 0.015 | 2 |
| chr11_7060948_C_T | NLRP14 | SER-LEU | 0.053 | 0.015 | 2 |
| chr11_7083610_T_A | NLRP14 | SER-THR | 0.053 | 0.015 | 2 |
| chr11_48328121_T_C | OR4S1 | MET-THR | 0.053 | 0.015 | 2 |
| chr11_72946279_T_C | P2RY2 | SER-PRO | 0.079 | 0.015 | 3 |
| chr11_108143456_C_G | ATM | PRO-ARG | 0.071 | 0.015 | 2 |
| chr11_115085467_A_C | CADM1 | ASP-GLU | 0.053 | 0.015 | 2 |
| chr12_57578673_G_A | LRP1 | ASP-ASN | 0.079 | 0.015 | 3 |
| chr12_108912178_G_A | FICD | none | 0.053 | 0.015 | 2 |
| chr12_120313953_C_T | CIT | GLY-GLU | 0.079 | 0.015 | 3 |
| chr12_122481879_A_C | BCL7A | ASN-THR | 0.079 | 0.015 | 3 |
| chr13_24823928_T_C | SPATA13 | VAL-ALA | 0.083 | 0.015 | 3 |
| chr13_31891743_G_A | B3GALTL | GLY-SER | 0.079 | 0.015 | 2 |
| chr14_51206145_A_T | NIN | SER-THR | 0.053 | 0.015 | 2 |
| chr15_34648647_T_A | C15orf55 | VAL-GLU | 0.111 | 0.015 | 3 |
| chr15_40660043_G_T | DISP2 | GLY-VAL | 0.088 | 0.015 | 3 |
| chr16_684579_C_T | C16orf13 | ALA-THR | 0.053 | 0.015 | 2 |
| chr17_4535314_G_A | ALOX15 | THR-MET | 0.083 | 0.015 | 2 |
| chr17_36483522_G_T | GPR179 | PRO-HIS | 0.083 | 0.015 | 2 |
| chr18_28604374_T_C | DSC3 | ASN-SER | 0.059 | 0.015 | 2 |
| chr19_372689_G_A | THEG | ARG-CYS | 0.053 | 0.015 | 2 |
| chr19_8201121_C_T | FBN3 | ARG-GLN | 0.053 | 0.015 | 2 |
| chr19_8203113_C_T | FBN3 | MET-ILE | 0.083 | 0.015 | 2 |
| chr19_10463118_G_C | TYK2 | PRO-ALA | 0.053 | 0.015 | 2 |
| chr19_19790178_A_G | ZNF101 | HIS-ARG | 0.079 | 0.015 | 3 |
| chr19_55144100_A_G | LILRB1 | THR-ALA | 0.056 | 0.015 | 2 |
| chr19_55144623_C_T | LILRB1 | THR-MET | 0.053 | 0.015 | 2 |
| chr19_57868483_G_A | ZNF304 | ALA-THR | 0.079 | 0.015 | 3 |
| chr22_37407109_G_C | TST | PRO-ALA | 0.053 | 0.015 | 2 |
| chr1_21267993_G_C | EIF4G3 | PRO-ALA | 0.053 | 0.016 | 2 |
| chr1_32842319_T_C | BSDC1 | ILE-VAL | 0.067 | 0.016 | 2 |
| chr1_33836164_G_A | PHC2 | ALA-VAL | 0.083 | 0.016 | 2 |
| chr1_35227162_C_T | GJB4 | ARG-CYS | 0.053 | 0.016 | 2 |
| chr1_35250457_C_T | GJB3 | ARG-TRP | 0.079 | 0.016 | 3 |
| chr1_52838992_A_G | ORC1 | MET-THR | 0.053 | 0.016 | 2 |
| chr1_116937809_A_G | ATP1A1 | ILE-VAL | 0.053 | 0.016 | 2 |
| chr1_117554216_G_A | CD101 | GLY-SER | 0.053 | 0.016 | 2 |
| chr1_155290231_T_C | FDPS | VAL-ALA | 0.053 | 0.016 | 2 |
| chr1_224482084_C_T | NVL | VAL-ILE | 0.105 | 0.016 | 3 |
| chr1_240370702_A_G | FMN2 | THR-ALA | 0.053 | 0.016 | 2 |
| chr2_103335646_A_G | MFSD9 | TRP-ARG | 0.056 | 0.016 | 2 |
| chr2_108875267_T_C | SULT1C3 | TYR-HIS | 0.053 | 0.016 | 2 |
| chr2_176829117_G_C | KIAA1715 | THR-SER | 0.053 | 0.016 | 2 |
| chr2_189932764_G_A | COL5A2 | PRO-SER | 0.053 | 0.016 | 2 |
| chr2_189940142_T_G | COL5A2 | MET-LEU | 0.056 | 0.016 | 2 |
| chr2_204073414_A_G | NBEAL1 | THR-ALA | 0.079 | 0.016 | 3 |
| chr2_216236712_T_C | FN1 | ILE-VAL | 0.053 | 0.016 | 2 |
| chr2_220404484_G_C | CHPF | ALA-GLY | 0.056 | 0.016 | 2 |
| chr3_52780205_A_G | NEK4 | PHE-LEU | 0.053 | 0.016 | 2 |
| chr3_184070901_C_T | CLCN2 | ARG-GLN | 0.053 | 0.016 | 2 |
| chr4_3526642_T_C | LRPAP1 | ASN-SER | 0.053 | 0.016 | 2 |
| chr4_57215969_C_T | AASDH | ASP-ASN | 0.053 | 0.016 | 2 |
| chr4_156832715_A_C | TDO2 | ASN-HIS | 0.056 | 0.016 | 2 |
| chr5_78135241_C_T | ARSB | SER-ASN | 0.083 | 0.016 | 3 |
| chr6_18139228_C_T | TPMT | ALA-THR | 0.053 | 0.016 | 2 |
| chr6_30671588_A_T | MDC1 | VAL-GLU | 0.079 | 0.016 | 3 |
| chr6_30671726_G_C | MDC1 | PRO-ARG | 0.079 | 0.016 | 3 |
| chr6_30673625_G_A | MDC1 | SER-PHE | 0.079 | 0.016 | 3 |
| chr6_30673662_G_C | MDC1 | PRO-ALA | 0.079 | 0.016 | 3 |
| chr6_30681477_G_A | MDC1 | ARG-CYS | 0.088 | 0.016 | 3 |
| chr7_66453476_A_G | SBDS | ILE-THR | 0.139 | 0.016 | 4 |
| chr7_127224872_G_A | GCC1 | ALA-VAL | 0.079 | 0.016 | 3 |
| chr7_135123060_G_C | CNOT4 | ALA-GLY | 0.053 | 0.016 | 2 |
| chr8_95479680_G_C | RAD54B | LEU-VAL | 0.056 | 0.016 | 2 |
| chr9_116224374_G_T | RGS3 | GLY-VAL | 0.053 | 0.016 | 2 |
| chr9_134390870_C_A | POMT1 | ASP-GLU | 0.053 | 0.016 | 2 |
| chr10_70987024_G_A | HKDC1 | ARG-GLN | 0.053 | 0.016 | 2 |
| chr10_135010635_G_A | KNDC1 | CYS-TYR | 0.115 | 0.016 | 3 |
| chr11_60906279_G_T | VPS37C | THR-ASN | 0.053 | 0.016 | 2 |
| chr11_67200812_C_T | RPS6KB2 | PRO-LEU | 0.105 | 0.016 | 4 |
| chr14_52509063_G_A | NID2 | PRO-SER | 0.056 | 0.016 | 2 |
| chr14_90752754_G_A | C14orf102 | ARG-CYS | 0.053 | 0.016 | 2 |
| chr15_31294702_G_T | TRPM1 | PRO-THR | 0.056 | 0.016 | 2 |
| chr15_75012987_G_T | CYP1A1 | THR-ASN | 0.094 | 0.016 | 3 |
| chr17_11543588_G_A | DNAH9 | none | 0.053 | 0.016 | 2 |
| chr17_36552190_A_G | SOCS7 | LYS-ARG | 0.079 | 0.016 | 3 |
| chr17_48594691_G_A | MYCBPAP | ARG-HIS | 0.053 | 0.016 | 2 |
| chr18_19996805_T_C | CTAGE1 | ILE-VAL | 0.154 | 0.016 | 3 |
| chr18_47363963_T_C | MYO5B | MET-VAL | 0.105 | 0.016 | 3 |
| chr18_50683727_T_A | DCC | none | 0.053 | 0.016 | 2 |
| chr18_56367823_A_G | MALT1 | ARG-GLY | 0.059 | 0.016 | 2 |
| chr19_41220029_G_A | ADCK4 | ARG-CYS | 0.184 | 0.016 | 6 |
| chr19_41235167_G_A | ITPKC | ARG-HIS | 0.053 | 0.016 | 2 |
| chr19_48525466_C_T | ELSPBP1 | PRO-LEU | 0.053 | 0.016 | 2 |
| chr20_60902402_G_A | LAMA5 | ARG-TRP | 0.125 | 0.016 | 4 |
| chr20_62325833_C_A | RTEL1 | PRO-HIS | 0.053 | 0.016 | 2 |
| chr21_43809092_C_T | TMPRSS3 | ALA-THR | 0.053 | 0.016 | 2 |
| chr22_32550293_C_T | C22orf42 | ARG-HIS | 0.079 | 0.016 | 3 |
| chr22_41574383_A_C | EP300 | GLN-PRO | 0.053 | 0.016 | 2 |
| chrX_14929375_G_A | MOSPD2 | SER-ASN | 0.111 | 0.016 | 4 |
| chrX_38147269_G_A | RPGR | THR-MET | 0.053 | 0.016 | 2 |
| chr1_55050353_G_A | ACOT11 | ARG-HIS | 0.056 | 0.017 | 2 |
| chr1_152282684_G_A | FLG | ARG-CYS | 0.079 | 0.017 | 3 |
| chr1_155257818_C_T | HCN3 | PRO-LEU | 0.056 | 0.017 | 2 |
| chr1_234565176_C_T | TARBP1 | VAL-MET | 0.079 | 0.017 | 3 |
| chr1_234582720_C_T | TARBP1 | GLY-ARG | 0.056 | 0.017 | 2 |
| chr2_98928757_C_T | VWA3B | THR-ILE | 0.053 | 0.017 | 2 |
| chr2_202626437_T_C | ALS2 | ILE-VAL | 0.079 | 0.017 | 3 |
| chr4_69795635_A_C | UGT2A3 | PHE-VAL | 0.053 | 0.017 | 2 |
| chr4_121719544_A_T | PRDM5 | SER-THR | 0.056 | 0.017 | 2 |
| chr5_135288632_A_G | LECT2 | ILE-THR | 0.053 | 0.017 | 2 |
| chr6_88140811_C_A | C6orf165 | ALA-GLU | 0.056 | 0.017 | 2 |
| chr7_30961790_G_A | AQP1 | GLY-ASP | 0.056 | 0.017 | 2 |
| chr7_142655008_G_A | KEL | THR-MET | 0.053 | 0.017 | 2 |
| chr7_148950895_C_T | ZNF212 | HIS-TYR | 0.077 | 0.017 | 2 |
| chr8_65527669_C_T | CYP7B1 | ARG-HIS | 0.053 | 0.017 | 2 |
| chr8_86021932_T_A | LRRCC1 | HIS-GLN | 0.107 | 0.017 | 3 |
| chr9_123875921_C_G | CNTRL | LEU-VAL | 0.053 | 0.017 | 2 |
| chr9_139297266_C_T | SDCCAG3 | VAL-ILE | 0.053 | 0.017 | 2 |
| chr10_116702425_A_C | TRUB1 | GLU-ALA | 0.053 | 0.017 | 2 |
| chr11_7324584_G_A | SYT9 | VAL-MET | 0.053 | 0.017 | 2 |
| chr11_66358274_A_C | CCDC87 | LEU-ARG | 0.053 | 0.017 | 2 |
| chr11_67809268_C_T | TCIRG1 | ARG-TRP | 0.056 | 0.017 | 2 |
| chr11_108357137_G_A | KDELC2 | PRO-LEU | 0.079 | 0.017 | 3 |
| chr12_113867073_G_C | SDSL | GLY-ALA | 0.053 | 0.017 | 2 |
| chr13_86370571_G_A | SLITRK6 | LEU-PHE | 0.079 | 0.017 | 3 |
| chr14_72169130_T_C | SIPA1L1 | ILE-THR | 0.053 | 0.017 | 2 |
| chr16_10775857_C_G | TEKT5 | ASP-HIS | 0.059 | 0.017 | 2 |
| chr16_27480797_C_T | GTF3C1 | ARG-HIS | 0.053 | 0.017 | 2 |
| chr16_71220755_A_G | HYDIN | MET-THR | 0.079 | 0.017 | 3 |
| chr17_36997503_T_C | C17orf98 | GLN-ARG | 0.053 | 0.017 | 2 |
| chr17_74622774_T_C | ST6GALNAC1 | ILE-VAL | 0.053 | 0.017 | 2 |
| chr19_11978828_G_T | ZNF439 | ARG-ILE | 0.053 | 0.017 | 2 |
| chr19_17108094_C_T | CPAMD8 | VAL-ILE | 0.100 | 0.017 | 2 |
| chr19_35510304_C_T | GRAMD1A | ARG-TRP | 0.059 | 0.017 | 2 |
| chr19_55107854_G_T | LILRA1 | VAL-LEU | 0.053 | 0.017 | 2 |
| chr20_7964476_T_C | TMX4 | TYR-CYS | 0.083 | 0.017 | 3 |
| chrX_53654402_T_C | HUWE1 | ASN-SER | 0.053 | 0.017 | 2 |
| chr1_12009911_G_A | PLOD1 | ALA-THR | 0.053 | 0.018 | 2 |
| chr1_22852880_G_C | ZBTB40 | GLU-ASP | 0.053 | 0.018 | 2 |
| chr1_25572984_T_G | C1orf63 | ARG-SER | 0.053 | 0.018 | 2 |
| chr1_40775937_G_A | COL9A2 | THR-MET | 0.053 | 0.018 | 2 |
| chr1_48825355_G_A | SPATA6 | ARG-TRP | 0.053 | 0.018 | 2 |
| chr1_74648329_G_A | LRRIQ3 | HIS-TYR | 0.053 | 0.018 | 2 |
| chr1_152281317_G_T | FLG | ASP-GLU | 0.079 | 0.018 | 3 |
| chr1_152285181_G_T | FLG | HIS-GLN | 0.079 | 0.018 | 3 |
| chr1_155640115_C_T | YY1AP1 | ASP-ASN | 0.083 | 0.018 | 3 |
| chr2_15519924_C_T | NBAS | ARG-HIS | 0.105 | 0.018 | 4 |
| chr2_48873925_A_G | GTF2A1L,STON1-GTF2A1L | TYR-CYS | 0.105 | 0.018 | 3 |
| chr2_217543728_G_A | IGFBP5 | ARG-TRP | 0.053 | 0.018 | 2 |
| chr2_220072431_C_A | ZFAND2B | PRO-HIS | 0.053 | 0.018 | 2 |
| chr2_239161957_C_T | PER2 | VAL-ILE | 0.053 | 0.018 | 2 |
| chr3_10276185_C_G | IRAK2 | LEU-VAL | 0.053 | 0.018 | 2 |
| chr4_39448586_C_T | KLB | ALA-VAL | 0.107 | 0.018 | 2 |
| chr5_94749787_C_T | FAM81B | GLN-stop | 0.053 | 0.018 | 2 |
| chr5_126250812_C_T | 3-Mar | ARG-GLN | 0.059 | 0.018 | 2 |
| chr5_137486640_A_T | BRD8 | CYS-SER | 0.053 | 0.018 | 2 |
| chr5_159659262_G_A | FABP6 | MET-ILE | 0.053 | 0.018 | 2 |
| chr6_29910660_A_G | HLA-A | GLN-ARG | 0.111 | 0.018 | 4 |
| chr6_122773119_T_C | SERINC1 | SER-GLY | 0.079 | 0.018 | 3 |
| chr7_123593764_T_C | SPAM1 | VAL-ALA | 0.059 | 0.018 | 2 |
| chr8_81426196_C_A | ZBTB10 | ALA-GLU | 0.111 | 0.018 | 2 |
| chr8_144557739_G_A | ZC3H3 | ARG-CYS | 0.083 | 0.018 | 2 |
| chr8_145154222_G_A | SHARPIN | PRO-SER | 0.079 | 0.018 | 3 |
| chr9_18928210_G_A | FAM154A | ALA-VAL | 0.053 | 0.018 | 2 |
| chr9_18928441_T_C | FAM154A | LYS-ARG | 0.053 | 0.018 | 2 |
| chr9_18928536_G_C | FAM154A | CYS-TRP | 0.053 | 0.018 | 2 |
| chr10_82126600_G_A | DYDC2 | GLY-SER | 0.053 | 0.018 | 2 |
| chr10_103988265_G_A | ELOVL3 | ASP-ASN | 0.053 | 0.018 | 2 |
| chr10_118351414_A_G | PNLIPRP1 | ASN-ASP | 0.053 | 0.018 | 2 |
| chr11_55033110_A_G | TRIM48 | GLU-GLY | 0.094 | 0.018 | 3 |
| chr11_59807978_G_A | PLAC1L | GLY-SER | 0.053 | 0.018 | 2 |
| chr11_71276655_G_A | KRTAP5-10 | GLY-ARG | 0.079 | 0.018 | 3 |
| chr12_333193_C_T | SLC6A13 | VAL-ILE | 0.053 | 0.018 | 2 |
| chr12_7985318_C_A | SLC2A14 | none | 0.079 | 0.018 | 2 |
| chr12_64485153_G_A | SRGAP1 | VAL-ILE | 0.079 | 0.018 | 3 |
| chr12_93139348_A_G | PLEKHG7 | ASP-GLY | 0.132 | 0.018 | 4 |
| chr14_57947421_G_A | C14orf105 | GLN-stop | 0.053 | 0.018 | 2 |
| chr14_92922782_G_A | SLC24A4 | SER-ASN | 0.053 | 0.018 | 2 |
| chr15_86122875_A_C | AKAP13 | LYS-GLN | 0.079 | 0.018 | 3 |
| chr16_3299468_C_T | MEFV | ARG-GLN | 0.132 | 0.018 | 5 |
| chr16_30492823_C_T | ITGAL | ARG-TRP | 0.083 | 0.018 | 3 |
| chr16_31473275_A_G | ARMC5 | ILE-VAL | 0.053 | 0.018 | 2 |
| chr16_57732012_G_A | CCDC135 | ASP-ASN | 0.053 | 0.018 | 2 |
| chr16_81209247_C_T | PKD1L2 | ARG-HIS | 0.053 | 0.018 | 2 |
| chr16_90126993_A_G | PRDM7 | LEU-PRO | 0.105 | 0.018 | 4 |
| chr17_15532147_G_A | TRIM16 | ARG-TRP | 0.053 | 0.018 | 2 |
| chr17_38715186_T_C | CCR7 | MET-VAL | 0.053 | 0.018 | 2 |
| chr17_59489707_C_T | C17orf82 | ALA-VAL | 0.111 | 0.018 | 4 |
| chr18_25532304_T_C | CDH2 | ASN-SER | 0.105 | 0.018 | 4 |
| chr19_4359191_C_T | MPND | PRO-LEU | 0.053 | 0.018 | 2 |
| chr19_38229926_G_A | ZNF573 | ARG-TRP | 0.105 | 0.018 | 4 |
| chr19_50251422_C_T | TSKS | GLU-LYS | 0.053 | 0.018 | 2 |
| chr22_23915620_C_T | IGLL1 | GLY-SER | 0.079 | 0.018 | 3 |
| chrX_31986607_G_A | DMD | ARG-TRP | 0.079 | 0.018 | 3 |
| chrX_101970316_A_C | GPRASP2,ARMCX5-GPRASP2 | ARG-SER | 0.053 | 0.018 | 2 |
| chr1_53569230_T_A | SLC1A7 | LYS-MET | 0.056 | 0.019 | 2 |
| chr1_104117921_G_A | AMY2B | GLY-ARG | 0.105 | 0.019 | 4 |
| chr1_186275564_C_T | PRG4 | THR-MET | 0.053 | 0.019 | 2 |
| chr1_202710733_C_G | KDM5B | GLU-GLN | 0.053 | 0.019 | 2 |
| chr1_207851554_C_T | CR1L | ARG-CYS | 0.143 | 0.019 | 2 |
| chr1_248458718_T_C | OR2T12 | ARG-GLY | 0.079 | 0.019 | 3 |
| chr2_21233999_T_C | APOB | ASN-SER | 0.053 | 0.019 | 2 |
| chr2_223085955_G_T | PAX3 | THR-LYS | 0.053 | 0.019 | 2 |
| chr4_3148570_G_A | HTT | VAL-ILE | 0.079 | 0.019 | 3 |
| chr4_146077132_C_T | OTUD4 | ALA-THR | 0.053 | 0.019 | 2 |
| chr5_102338811_A_G | PAM | ASP-GLY | 0.118 | 0.019 | 4 |
| chr5_102537285_A_G | PPIP5K2 | SER-GLY | 0.079 | 0.019 | 3 |
| chr7_94540527_G_A | PPP1R9A | ASP-ASN | 0.132 | 0.019 | 5 |
| chr7_134849209_C_T | TMEM140 | PRO-SER | 0.132 | 0.019 | 4 |
| chr8_10466089_G_C | RP1L1 | ALA-GLY | 0.053 | 0.019 | 2 |
| chr8_10466161_C_T | RP1L1 | GLY-ASP | 0.053 | 0.019 | 2 |
| chr11_1298430_C_A | TOLLIP | ALA-SER | 0.071 | 0.019 | 2 |
| chr11_7083620_T_C | NLRP14 | LEU-SER | 0.053 | 0.019 | 2 |
| chr11_7694002_G_A | CYB5R2 | PRO-SER | 0.053 | 0.019 | 2 |
| chr11_45937306_C_T | PEX16 | VAL-MET | 0.079 | 0.019 | 3 |
| chr11_60197299_G_A | MS4A5 | GLY-GLU | 0.091 | 0.019 | 2 |
| chr11_64599139_C_T | CDC42BPG | ALA-THR | 0.059 | 0.019 | 2 |
| chr11_68174189_G_A | LRP5 | VAL-MET | 0.056 | 0.019 | 2 |
| chr14_88452945_G_A | GALC | none | 0.053 | 0.019 | 2 |
| chr19_17108136_G_A | CPAMD8 | ARG-TRP | 0.143 | 0.019 | 3 |
| chr19_31039669_C_T | ZNF536 | ALA-VAL | 0.105 | 0.019 | 4 |
| chr20_32266134_C_T | E2F1 | GLY-SER | 0.079 | 0.019 | 3 |
| chr21_45820196_C_T | TRPM2 | ARG-CYS | 0.053 | 0.019 | 2 |
| chr1_29320013_G_A | EPB41 | VAL-ILE | 0.053 | 0.020 | 2 |
| chr1_82456585_G_A | LPHN2 | ARG-LYS | 0.079 | 0.020 | 3 |
| chr1_152191696_G_C | HRNR | SER-ARG | 0.079 | 0.020 | 3 |
| chr1_152193605_C_T | HRNR | GLY-ASP | 0.079 | 0.020 | 3 |
| chr1_152281691_G_A | FLG | ARG-TRP | 0.079 | 0.020 | 3 |
| chr1_152281745_G_T | FLG | GLN-LYS | 0.079 | 0.020 | 3 |
| chr1_152282794_G_A | FLG | THR-ILE | 0.079 | 0.020 | 3 |
| chr1_160011512_G_A | KCNJ10 | ARG-CYS | 0.079 | 0.020 | 3 |
| chr1_217804767_A_G | SPATA17 | ASN-SER | 0.105 | 0.020 | 4 |
| chr1_248309159_C_A | OR2M5 | ALA-ASP | 0.053 | 0.020 | 2 |
| chr2_110959026_G_T | NPHP1 | PRO-THR | 0.091 | 0.020 | 2 |
| chr2_215813331_C_T | ABCA12 | ASP-ASN | 0.056 | 0.020 | 2 |
| chr3_193031875_C_T | ATP13A5 | ALA-THR | 0.053 | 0.020 | 2 |
| chr4_3137674_G_A | HTT | GLY-ARG | 0.056 | 0.020 | 2 |
| chr5_118480316_G_A | DMXL1 | SER-ASN | 0.105 | 0.020 | 4 |
| chr6_73904557_C_T | KCNQ5 | PRO-LEU | 0.079 | 0.020 | 3 |
| chr6_131179302_C_G | EPB41L2 | GLU-GLN | 0.053 | 0.020 | 2 |
| chr6_143929450_G_A | PHACTR2 | ASP-ASN | 0.053 | 0.020 | 2 |
| chr6_159398803_C_T | RSPH3 | GLU-LYS | 0.053 | 0.020 | 2 |
| chr6_159401898_C_T | RSPH3 | ARG-GLN | 0.053 | 0.020 | 2 |
| chr7_92132493_T_C | PEX1 | ILE-MET | 0.059 | 0.020 | 2 |
| chr9_105767349_g_T | CYLC2 | ASP-TYR | 0.071 | 0.020 | 2 |
| chr10_129690837_G_T | CLRN3 | PRO-GLN | 0.056 | 0.020 | 2 |
| chr12_52960884_C_T | KRT74 | ALA-THR | 0.077 | 0.020 | 2 |
| chr14_21109745_C_G | OR6S1 | VAL-LEU | 0.053 | 0.020 | 2 |
| chr15_93557954_G_C | CHD2 | GLY-ALA | 0.053 | 0.020 | 2 |
| chr15_98512431_C_T | ARRDC4 | THR-MET | 0.079 | 0.020 | 3 |
| chr15_99762041_C_T | TTC23 | ARG-HIS | 0.079 | 0.020 | 3 |
| chr16_15870032_A_G | MYH11 | none | 0.053 | 0.020 | 2 |
| chr16_27373980_C_T | IL4R | SER-LEU | 0.053 | 0.020 | 2 |
| chr19_7584483_A_C | ZNF358 | LYS-GLN | 0.053 | 0.020 | 2 |
| chr19_14578707_G_A | PKN1 | ARG-GLN | 0.063 | 0.020 | 2 |
| chr19_20044932_G_A | ZNF93 | VAL-ILE | 0.056 | 0.020 | 2 |
| chr19_41356246_C_T | CYP2A6 | SER-ASN | 0.053 | 0.020 | 2 |
| chr19_44932521_C_T | ZNF229 | ARG-GLN | 0.079 | 0.020 | 3 |
| chr19_55525818_C_T | GP6 | GLY-SER | 0.079 | 0.020 | 3 |
| chr19_57326970_T_C | PEG3,ZIM2 | ASN-SER | 0.053 | 0.020 | 2 |
| chr20_60768573_C_T | GTPBP5 | ARG-TRP | 0.053 | 0.020 | 2 |
| chr21_38563639_C_T | TTC3 | PRO-SER | 0.077 | 0.020 | 2 |
| chr22_43218397_T_C | ARFGAP3 | SER-GLY | 0.053 | 0.020 | 2 |
| chrX_23723711_C_G | ACOT9 | GLU-GLN | 0.059 | 0.020 | 2 |
| chrX_38268220_A_G | OTC | GLN-ARG | 0.053 | 0.020 | 2 |
| chr1_63872032_T_C | ALG6 | TYR-HIS | 0.079 | 0.021 | 3 |
| chr1_152193162_C_T | HRNR | GLY-SER | 0.079 | 0.021 | 3 |
| chr2_27167617_G_A | DPYSL5 | ALA-THR | 0.158 | 0.021 | 6 |
| chr2_79313990_C_T | REG1B | ARG-HIS | 0.053 | 0.021 | 2 |
| chr2_102968212_A_G | IL1RL1 | GLN-ARG | 0.316 | 0.021 | 10 |
| chr2_162904013_T_C | DPP4 | none | 0.071 | 0.021 | 2 |
| chr3_38167095_A_G | ACAA1 | VAL-ALA | 0.053 | 0.021 | 2 |
| chr3_74334560_C_T | CNTN3 | ARG-GLN | 0.053 | 0.021 | 2 |
| chr3_186572089_T_C | ADIPOQ | TYR-HIS | 0.053 | 0.021 | 2 |
| chr4_5016883_G_A | CYTL1 | ARG-CYS | 0.053 | 0.021 | 2 |
| chr4_38987965_C_T | TMEM156 | VAL-ILE | 0.083 | 0.021 | 3 |
| chr4_75248505_G_T | EREG | CYS-PHE | 0.053 | 0.021 | 2 |
| chr4_96091414_C_T | UNC5C | ALA-THR | 0.053 | 0.021 | 2 |
| chr5_35705844_A_C | SPEF2 | LYS-GLN | 0.125 | 0.021 | 2 |
| chr5_101834469_G_A | SLCO6A1 | ALA-VAL | 0.079 | 0.021 | 3 |
| chr5_150666946_C_T | SLC36A3 | ARG-HIS | 0.079 | 0.021 | 3 |
| chr6_33260215_C_T | RGL2 | GLY-GLU | 0.053 | 0.021 | 2 |
| chr7_151949735_T_C | MLL3 | ILE-MET | 0.059 | 0.021 | 2 |
| chr8_10464604_T_C | RP1L1 | HIS-ARG | 0.132 | 0.021 | 5 |
| chr8_17739639_C_A | FGL1 | ARG-LEU | 0.053 | 0.021 | 2 |
| chr8_17743051_A_T | FGL1 | PHE-ILE | 0.053 | 0.021 | 2 |
| chr8_32611970_G_T | NRG1 | VAL-LEU | 0.053 | 0.021 | 2 |
| chr8_130789767_G_A | GSDMC | PRO-SER | 0.053 | 0.021 | 2 |
| chr8_144804299_T_C | MAPK15 | SER-PRO | 0.053 | 0.021 | 2 |
| chr8_144808926_C_T | FAM83H | GLY-GLU | 0.056 | 0.021 | 2 |
| chr9_137642654_G_A | COL5A1 | GLY-SER | 0.083 | 0.021 | 2 |
| chr10_7774358_C_G | ITIH2 | LEU-VAL | 0.111 | 0.021 | 4 |
| chr10_123996970_G_A | TACC2 | GLY-ARG | 0.053 | 0.021 | 2 |
| chr10_129907610_G_A | MKI67 | ARG-TRP | 0.053 | 0.021 | 2 |
| chr11_5878836_T_A | OR52E8 | ILE-PHE | 0.053 | 0.021 | 2 |
| chr12_1137217_A_G | ERC1 | SER-GLY | 0.053 | 0.021 | 2 |
| chr12_7288432_A_G | CLSTN3 | SER-GLY | 0.053 | 0.021 | 2 |
| chr12_39735348_C_A | KIF21A | GLY-VAL | 0.053 | 0.021 | 2 |
| chr12_52841174_C_T | KRT6B | GLY-SER | 0.056 | 0.021 | 2 |
| chr12_69744014_C_A | LYZ | THR-ASN | 0.105 | 0.021 | 4 |
| chr12_93181768_C_A | EEA1 | ALA-SER | 0.056 | 0.021 | 2 |
| chr15_31295151_T_G | TRPM1 | ASN-THR | 0.053 | 0.021 | 2 |
| chr16_16276292_T_C | ABCC6 | ILE-VAL | 0.053 | 0.021 | 2 |
| chr16_81916912_A_G | PLCG2 | HIS-ARG | 0.053 | 0.021 | 2 |
| chr16_89927151_C_T | SPIRE2 | SER-LEU | 0.067 | 0.021 | 2 |
| chr17_1540027_C_T | SCARF1 | ALA-THR | 0.067 | 0.021 | 2 |
| chr17_37792090_G_A | PPP1R1B | ARG-HIS | 0.056 | 0.021 | 2 |
| chr17_40263400_C_T | DHX58 | ARG-GLN | 0.056 | 0.021 | 2 |
| chr19_18376518_T_A | KIAA1683 | GLN-LEU | 0.079 | 0.021 | 3 |
| chr19_18897440_T_C | COMP | ASN-ASP | 0.053 | 0.021 | 2 |
| chr19_35232987_A_C | ZNF181 | ARG-SER | 0.125 | 0.021 | 3 |
| chr19_38782485_G_C | SPINT2 | VAL-LEU | 0.056 | 0.021 | 2 |
| chr19_54783693_C_T | LILRB2 | ARG-HIS | 0.053 | 0.021 | 2 |
| chr20_5843952_G_A | C20orf196 | ARG-GLN | 0.105 | 0.021 | 4 |
| chr1_39340558_G_A | GJA9 | PRO-SER | 0.053 | 0.022 | 2 |
| chr1_161132821_C_A | USP21 | PRO-THR | 0.105 | 0.022 | 3 |
| chr1_183099560_G_A | LAMC1 | ARG-GLN | 0.053 | 0.022 | 2 |
| chr1_206224635_G_C | AVPR1B | LYS-ASN | 0.083 | 0.022 | 3 |
| chr2_165578602_C_T | COBLL1 | VAL-MET | 0.132 | 0.022 | 4 |
| chr2_209179939_A_G | PIKFYVE | MET-VAL | 0.053 | 0.022 | 2 |
| chr2_239237388_G_A | TRAF3IP1 | ARG-GLN | 0.053 | 0.022 | 2 |
| chr3_15686693_G_C | BTD | ASP-HIS | 0.053 | 0.022 | 2 |
| chr3_48623124_G_A | COL7A1 | PRO-LEU | 0.079 | 0.022 | 3 |
| chr4_5755516_T_A | EVC | PHE-LEU | 0.059 | 0.022 | 2 |
| chr4_123171659_T_A | KIAA1109 | ASP-GLU | 0.053 | 0.022 | 2 |
| chr4_123179900_C_T | KIAA1109 | ARG-TRP | 0.105 | 0.022 | 4 |
| chr5_5182286_C_T | ADAMTS16 | PRO-SER | 0.059 | 0.022 | 2 |
| chr5_70858268_A_G | BDP1 | ASN-SER | 0.083 | 0.022 | 3 |
| chr5_140558628_T_C | PCDHB8 | VAL-ALA | 0.079 | 0.022 | 3 |
| chr6_32487242_G_A | HLA-DRB5 | THR-ILE | 0.133 | 0.022 | 3 |
| chr6_44270870_C_T | AARS2 | VAL-MET | 0.079 | 0.022 | 3 |
| chr6_74497102_G_A | CD109 | GLY-GLU | 0.079 | 0.022 | 2 |
| chr6_149903597_A_G | C6orf72 | LYS-GLU | 0.056 | 0.022 | 2 |
| chr7_72985148_C_T | TBL2 | VAL-ILE | 0.053 | 0.022 | 2 |
| chr7_73279659_G_A | WBSCR28 | GLY-SER | 0.053 | 0.022 | 2 |
| chr9_21217166_T_C | IFNA16 | ILE-VAL | 0.053 | 0.022 | 2 |
| chr10_7772035_A_G | ITIH2 | ASN-SER | 0.105 | 0.022 | 4 |
| chr11_408174_G_T | SIGIRR | SER-TYR | 0.079 | 0.022 | 2 |
| chr11_419706_T_C | ANO9 | THR-ALA | 0.079 | 0.022 | 2 |
| chr11_7507182_A_G | OLFML1 | THR-ALA | 0.105 | 0.022 | 4 |
| chr11_57947163_G_A | OR9Q1 | ALA-THR | 0.079 | 0.022 | 3 |
| chr11_59224765_A_C | OR4D6 | ASP-ALA | 0.053 | 0.022 | 2 |
| chr11_64813685_G_C | NAALADL1 | LEU-VAL | 0.079 | 0.022 | 3 |
| chr12_18841115_G_A | PLCZ1 | SER-LEU | 0.105 | 0.022 | 4 |
| chr14_64604595_G_A | SYNE2 | GLU-LYS | 0.053 | 0.022 | 2 |
| chr15_51350287_T_G | TNFAIP8L3 | ASN-HIS | 0.139 | 0.022 | 4 |
| chr16_1272275_C_T | TPSG1 | ARG-HIS | 0.077 | 0.022 | 2 |
| chr16_3299586_G_A | MEFV | PRO-SER | 0.132 | 0.022 | 5 |
| chr16_4920335_A_G | UBN1 | TYR-CYS | 0.053 | 0.022 | 2 |
| chr16_19548030_T_A | CCP110 | PHE-ILE | 0.056 | 0.022 | 2 |
| chr16_49671177_T_C | ZNF423 | ASN-SER | 0.053 | 0.022 | 2 |
| chr18_43204739_C_T | SLC14A2 | THR-ILE | 0.079 | 0.022 | 3 |
| chr19_2936535_G_A | ZNF77 | GLN-stop | 0.158 | 0.022 | 6 |
| chr19_10426597_T_C | FDX1L | ARG-GLY | 0.100 | 0.022 | 3 |
| chr19_17108052_C_T | CPAMD8 | VAL-MET | 0.063 | 0.022 | 2 |
| chr19_19745479_C_T | GMIP | ASP-ASN | 0.067 | 0.022 | 2 |
| chr19_42213670_G_A | CEACAM5 | ALA-THR | 0.079 | 0.022 | 3 |
| chr19_52034506_C_T | SIGLEC6 | ARG-GLN | 0.053 | 0.022 | 2 |
| chr19_52520372_C_T | ZNF614 | GLY-GLU | 0.125 | 0.022 | 4 |
| chr20_24964558_T_C | C20orf3 | ILE-VAL | 0.079 | 0.022 | 3 |
| chr20_25282967_C_T | ABHD12 | ALA-THR | 0.053 | 0.022 | 2 |
| chr22_44031042_T_C | EFCAB6 | THR-ALA | 0.053 | 0.022 | 2 |
| chrX_16847765_A_G | TXLNG | ILE-VAL | 0.079 | 0.022 | 3 |
| chrX_92964617_G_A | FAM133A | GLU-LYS | 0.059 | 0.022 | 2 |
| chrX_152721728_T_C | HAUS7 | THR-ALA | 0.079 | 0.022 | 3 |
| chr1_16354394_C_T | CLCNKA | ALA-VAL | 0.118 | 0.023 | 4 |
| chr1_158624528_G_T | SPTA1 | ALA-ASP | 0.105 | 0.023 | 4 |
| chr1_180905263_C_T | KIAA1614 | ARG-TRP | 0.053 | 0.023 | 2 |
| chr1_221053574_a_C | HLX | GLN-HIS | 0.133 | 0.023 | 3 |
| chr1_241767708_C_T | OPN3 | VAL-ILE | 0.053 | 0.023 | 2 |
| chr2_98809432_T_C | VWA3B | ILE-THR | 0.105 | 0.023 | 4 |
| chr2_182780874_C_T | SSFA2 | PRO-LEU | 0.053 | 0.023 | 2 |
| chr3_172835392_A_T | SPATA16 | SER-THR | 0.079 | 0.023 | 3 |
| chr5_115338958_G_T | AQPEP | VAL-PHE | 0.077 | 0.023 | 2 |
| chr5_132561468_C_A | FSTL4 | GLU-ASP | 0.132 | 0.023 | 5 |
| chr5_149003626_G_A | ARHGEF37 | ALA-THR | 0.079 | 0.023 | 3 |
| chr6_29142347_A_T | OR2J2 | LYS-MET | 0.079 | 0.023 | 3 |
| chr7_7278447_T_A | C1GALT1 | ILE-ASN | 0.063 | 0.023 | 2 |
| chr7_37923923_T_C | TXNDC3 | ILE-THR | 0.091 | 0.023 | 2 |
| chr9_215296_A_G | DOCK8,C9orf66 | VAL-ALA | 0.063 | 0.023 | 2 |
| chr9_19290794_A_C | DENND4C | ILE-LEU | 0.083 | 0.023 | 3 |
| chr9_33385241_C_A | AQP7 | GLY-VAL | 0.053 | 0.023 | 2 |
| chr9_136522274_C_T | DBH | ARG-CYS | 0.053 | 0.023 | 2 |
| chr10_25312895_C_A | THNSL1 | ALA-GLU | 0.083 | 0.023 | 3 |
| chr10_85956268_C_A | CDHR1 | HIS-GLN | 0.053 | 0.023 | 2 |
| chr11_430339_C_G | ANO9 | VAL-LEU | 0.056 | 0.023 | 2 |
| chr12_13366504_G_A | EMP1 | SER-ASN | 0.105 | 0.023 | 4 |
| chr12_52760957_C_T | KRT85 | ARG-HIS | 0.053 | 0.023 | 2 |
| chr12_91366649_G_C | EPYC | SER-CYS | 0.056 | 0.023 | 2 |
| chr17_47284735_T_C | GNGT2 | GLN-ARG | 0.053 | 0.023 | 2 |
| chr17_74162548_C_T | RNF157 | GLY-ARG | 0.053 | 0.023 | 2 |
| chr18_74580780_C_T | ZNF236 | SER-LEU | 0.105 | 0.023 | 4 |
| chr19_7992976_T_C | TIMM44 | ILE-VAL | 0.056 | 0.023 | 2 |
| chr19_8197958_C_T | FBN3 | VAL-ILE | 0.118 | 0.023 | 3 |
| chr19_43763144_C_T | PSG9 | GLY-ARG | 0.079 | 0.023 | 2 |
| chr20_17602571_A_G | RRBP1 | CYS-ARG | 0.105 | 0.023 | 3 |
| chr21_45945648_G_C | TSPEAR | HIS-GLN | 0.079 | 0.023 | 3 |
| chr22_42526763_C_T | CYP2D6 | VAL-MET | 0.063 | 0.023 | 2 |
| chrX_49113312_G_A | FOXP3 | none | 0.079 | 0.023 | 3 |
| chr1_33944993_A_G | ZSCAN20 | LYS-ARG | 0.053 | 0.024 | 2 |
| chr1_43232504_C_A | LEPRE1 | ALA-SER | 0.063 | 0.024 | 2 |
| chr1_43675499_C_T | WDR65 | SER-LEU | 0.079 | 0.024 | 3 |
| chr1_43804340_G_A | MPL | VAL-MET | 0.053 | 0.024 | 2 |
| chr1_110019439_A_G | SYPL2 | GLU-GLY | 0.053 | 0.024 | 2 |
| chr1_201079344_G_C | CACNA1S | ALA-GLY | 0.053 | 0.024 | 2 |
| chr2_241622034_G_C | AQP12B | THR-SER | 0.125 | 0.024 | 4 |
| chr3_105264176_G_T | ALCAM | MET-ILE | 0.053 | 0.024 | 2 |
| chr4_4322670_C_T | ZBTB49 | ALA-VAL | 0.111 | 0.024 | 4 |
| chr5_102537298_C_T | PPIP5K2 | THR-MET | 0.056 | 0.024 | 2 |
| chr5_122364538_T_C | PPIC | LYS-ARG | 0.083 | 0.024 | 3 |
| chr5_171777495_G_T | SH3PXD2B | PRO-GLN | 0.056 | 0.024 | 2 |
| chr6_26093141_G_A | HFE | CYS-TYR | 0.105 | 0.024 | 4 |
| chr6_108214694_C_T | SEC63 | VAL-ILE | 0.053 | 0.024 | 2 |
| chr7_2289586_G_A | NUDT1 | VAL-MET | 0.053 | 0.024 | 2 |
| chr9_91616843_G_A | S1PR3 | ARG-GLN | 0.053 | 0.024 | 2 |
| chr9_95396712_G_A | IPPK | LEU-PHE | 0.053 | 0.024 | 2 |
| chr9_120475602_C_T | TLR4 | THR-ILE | 0.059 | 0.024 | 2 |
| chr10_124358498_T_G | DMBT1 | ILE-MET | 0.167 | 0.024 | 2 |
| chr11_60703882_G_A | TMEM132A | VAL-MET | 0.053 | 0.024 | 2 |
| chr11_62397114_T_C | GANAB | MET-VAL | 0.105 | 0.024 | 3 |
| chr12_53453433_G_A | TENC1 | ALA-THR | 0.056 | 0.024 | 2 |
| chr13_38211105_T_C | TRPC4 | ILE-VAL | 0.105 | 0.024 | 4 |
| chr14_20837033_T_C | TEP1 | HIS-ARG | 0.158 | 0.024 | 6 |
| chr14_20846950_C_T | TEP1 | ARG-GLN | 0.105 | 0.024 | 4 |
| chr14_64604592_C_G | SYNE2 | PRO-ALA | 0.053 | 0.024 | 2 |
| chr16_816977_G_A | MSLN | GLY-GLU | 0.105 | 0.024 | 4 |
| chr16_15818842_A_G | MYH11,NDE1 | VAL-ALA | 0.053 | 0.024 | 2 |
| chr17_2202323_T_C | SMG6 | ASN-SER | 0.056 | 0.024 | 2 |
| chr17_11511480_G_A | DNAH9 | ARG-HIS | 0.071 | 0.024 | 2 |
| chr17_39525750_C_T | KRT33B | GLU-LYS | 0.053 | 0.024 | 2 |
| chr18_13681962_A_T | C18orf19 | TYR-ASN | 0.079 | 0.024 | 3 |
| chr19_38876202_C_T | GGN | SER-ASN | 0.053 | 0.024 | 2 |
| chr19_45297479_C_T | CBLC | PRO-SER | 0.053 | 0.024 | 2 |
| chr20_57244396_G_A | STX16 | ARG-GLN | 0.056 | 0.024 | 2 |
| chr22_50722167_T_C | PLXNB2 | THR-ALA | 0.059 | 0.024 | 2 |
| chrX_83141579_A_G | CYLC1 | HIS-ARG | 0.053 | 0.024 | 2 |
| chrX_151123384_G_A | GABRE | PRO-LEU | 0.059 | 0.024 | 2 |
| chr1_881918_G_A | NOC2L | SER-LEU | 0.083 | 0.025 | 3 |
| chr1_45800156_C_T | MUTYH | VAL-MET | 0.105 | 0.025 | 3 |
| chr1_186281400_C_T | TPR,PRG4 | THR-MET | 0.053 | 0.025 | 2 |
| chr2_69177269_C_A | GKN2 | ASP-TYR | 0.083 | 0.025 | 2 |
| chr2_102968211_C_A | IL1RL1 | GLN-LYS | 0.316 | 0.025 | 10 |
| chr2_160604514_C_T | 7-Mar | THR-MET | 0.088 | 0.025 | 3 |
| chr2_240982275_A_G | PRR21 | ILE-THR | 0.079 | 0.025 | 3 |
| chr4_1843324_C_T | LETM1 | ARG-HIS | 0.132 | 0.025 | 5 |
| chr6_28121278_A_G | ZNF192 | GLN-ARG | 0.053 | 0.025 | 2 |
| chr8_23002090_C_T | TNFRSF10D | ARG-HIS | 0.083 | 0.025 | 3 |
| chr9_116191205_A_C | C9orf43 | ASN-THR | 0.083 | 0.025 | 3 |
| chr10_54531242_G_A | MBL2 | ARG-CYS | 0.132 | 0.025 | 4 |
| chr10_97143826_T_C | SORBS1 | TYR-CYS | 0.132 | 0.025 | 4 |
| chr11_4598956_c_T | C11orf40 | TRP-stop | 0.056 | 0.025 | 2 |
| chr11_130332457_T_C | ADAMTS15 | TYR-HIS | 0.079 | 0.025 | 2 |
| chr12_31256517_C_T | DDX11 | ALA-VAL | 0.088 | 0.025 | 3 |
| chr12_52841179_T_C | KRT6B | TYR-CYS | 0.088 | 0.025 | 3 |
| chr12_121712301_C_T | CAMKK2 | SER-ASN | 0.056 | 0.025 | 2 |
| chr14_22038562_T_C | OR10G3 | TYR-CYS | 0.079 | 0.025 | 3 |
| chr15_41046883_C_A | FAM82A2 | GLN-HIS | 0.056 | 0.025 | 2 |
| chr16_31470886_T_A | ARMC5 | PHE-TYR | 0.115 | 0.025 | 3 |
| chr17_5271763_G_C | RABEP1 | MET-ILE | 0.132 | 0.025 | 4 |
| chr17_18653188_G_T | FBXW10 | ARG-LEU | 0.056 | 0.025 | 2 |
| chr17_67017930_T_C | ABCA9 | ASN-SER | 0.083 | 0.025 | 3 |
| chr17_73517536_G_A | TSEN54 | VAL-MET | 0.053 | 0.025 | 2 |
| chrX_153689893_G_A | PLXNA3 | ARG-GLN | 0.053 | 0.025 | 2 |

^1^ Gene ID, symbols, amino acid changes, patient and Genome 200 allele frequency are given starting from SNPS that are found in 2 or in **n** number of patients (n).
