# Supplementary material for: Discovery of Potential New Gene Variants and Inflammatory Cytokine Associations with Fibromyalgia Syndrome by Whole Exome Sequencing
Source: PLoS One. 2013 Jun 10;8(6):e65033. doi: 10.1371/journal.pone.0065033 (PMC3677902; doi:10.1371/journal.pone.0065033)
Supplement: Table S3 — Transmission analysis for mutation W32X of the C11orf40 gene. The genotypes (heterozygous or homozygous) for proband, mother and father are reported together with the analysis of transmitted (t(B)) or untransmitted (ut(C)) from parent to proband. P = 0.026. (DOCX) [file pone.0065033.s005.docx]

**Table S3. Transmission analysis for mutation W32X of the C11orf40 gene.** The genotypes (heterozygous or homozygous) for proband, mother and father are reported together with the analysis of transmitted (t(B)) or untransmitted (ut(C)) from parent to proband. P= 0.026.

| **Proband** | |  | **Mother** | |  | **Father** | |  | **Allele** | |
| --- | --- | --- | --- | --- | --- | --- | --- | --- | --- | --- |
| **ID** | **genotype** |  | **ID** | **genotype** |  | **ID** | **genotype** |  | **ut (C)** | **t (B)** |
| FMS23 | het |  | FMS22 | wt |  | FMS21 | het |  | 0 | 1 |
| FMS65 | het |  | FMS66 | het |  | FMS67 | wt |  | 0 | 1 |
| FMS82 | het |  | FMS83 | wt |  | FMS84 | het |  | 0 | 1 |
| FMS179 | het |  | FMS180 | wt |  | FMS181 | het |  | 0 | 1 |
| FMS203 | homo |  | FMS204 | het |  | FMS205 | het |  | 0 | 2 |
| FMS235 | het |  | FMS236 | homo |  | FMS237 | wt |  |  |  |
| FMS244 | het |  | FMS245 | het |  | FMS243 | wt |  | 0 | 1 |
| FMS248 | het |  | FMS247 | wt |  | FMS249 | het |  | 0 | 1 |
| FMS366 | het |  | FMS339 | wt |  | FMS365 | het |  | 0 | 1 |
| FMS411 | het |  | FMS464 | het |  | FMS463 | wt |  | 0 | 1 |
| FMS416 | het |  | FMS415 | wt |  | FMS414 | het |  | 0 | 1 |
| FMS512 | het |  | FMS511 | wt |  | FMS510 | het |  | 0 | 1 |
| FMS515 | het |  | FMS514 | het |  | FMS513 | wt |  | 0 | 1 |
| FMS521 | het |  | FMS520 | wt |  | FMS519 | het |  | 0 | 1 |
| FMS561 | het |  | FMS560 | wt |  | FMS559 | het |  | 0 | 1 |
| FMS603 | het |  | FMS602 | het |  | FMS601 | wt |  | 0 | 1 |
| FMS632 | het |  | FMS631 | het |  | FMS630 | wt |  | 0 | 1 |
| FMS635 | het |  | FMS634 | homo |  | FMS633 | wt |  |  |  |
| FMS708 | het |  | FMS710 | het |  | FMS709 | wt |  | 0 | 1 |
| FMS733 | het |  | FMS735 | wt |  | FMS734 | het |  | 0 | 1 |
| FMS116 | wt |  | FMS161 | wt |  | FMS115 | het |  | 1 | 0 |
| FMS122 | wt |  | FMS123 | het |  | FMS124 | wt |  | 1 | 0 |
| FMS176 | wt |  | FMS177 | het |  | FMS178 | wt |  | 1 | 0 |
| FMS232 | wt |  | FMS233 | het |  | FMS234 | wt |  | 1 | 0 |
| FMS398 | wt |  | FMS399 | wt |  | FMS433 | het |  | 1 | 0 |
| FMS470 | wt |  | FMS469 | wt |  | FMS465 | het |  | 1 | 0 |
| FMS691 | wt |  | FMS693 | wt |  | FMS692 | het |  | 1 | 0 |
| FMS743 | wt |  | FMS745 | het |  | FMS744 | wt |  | 1 | 0 |
| **Total** |  |  |  |  |  |  |  |  | **8** | **19** |

^1^ het= heterozygous, wt= wild type; ut= untransmitted, t= transmitted; P= 0.026.
