# Supplementary material for: Discovery of Potential New Gene Variants and Inflammatory Cytokine Associations with Fibromyalgia Syndrome by Whole Exome Sequencing
Source: PLoS One. 2013 Jun 10;8(6):e65033. doi: 10.1371/journal.pone.0065033 (PMC3677902; doi:10.1371/journal.pone.0065033)
Supplement: Table S4 — Transmission analysis for Q100X of ZNF77 gene. The genotypes (heterozygous or homozygous) for proband, mother and father are reported together with the analysis of transmitted (t(B)) or untransmitted (ut(C)) from parent to proband. P = 0.032. (DOCX) [file pone.0065033.s006.docx]

**Table S4. Transmission analysis for Q100X of ZNF77 gene.** The genotypes (heterozygous or homozygous) for proband, mother and father are reported together with the analysis of transmitted (t(B)) or untransmitted (ut(C)) from parent to proband. P= 0.032.

| **Proband** | | **Mother** | | **Father** | | **Allele** | |
| --- | --- | --- | --- | --- | --- | --- | --- |
| **ID** | **genotype** | **ID** | **genotype** | **ID** | **genotype** | **ut(C)** | **t(B)** |
| FMS65 | het | FMS66 | wt | FMS67 | het | 0 | 1 |
| FMS68 | het | FMS69 | het | FMS71 | wt | 0 | 1 |
| FMS82 | het | FMS83 | het | FMS84 | wt | 0 | 1 |
| FMS93 | het | FMS94 | het | FMS95 | wt | 0 | 1 |
| FMS136 | het | FMS134 | wt | FMS135 | het | 0 | 1 |
| FMS189 | het | FMS190 | het | FMS191 | wt | 0 | 1 |
| FMS268 | het | FMS273 | het | FMS270 | wt | 0 | 1 |
| FMS340 | het | FMS339 | het | FMS365 | wt | 0 | 1 |
| FMS361 | het | FMS362 | het | FMS363 | wt | 0 | 1 |
| FMS398 | het | FMS399 | wt | FMS433 | het | 0 | 1 |
| FMS411 | het | FMS464 | het | FMS463 | wt | 0 | 1 |
| FMS483 | het | FMS484 | wt | FMS453 | het | 0 | 1 |
| FMS501 | het | FMS503 | wt | FMS502 | het | 0 | 1 |
| FMS553 | het | FMS552 | het | FMS551 | wt | 0 | 1 |
| FMS561 | het | FMS560 | het | FMS559 | wt | 0 | 1 |
| FMS603 | het | FMS602 | het | FMS601 | wt | 0 | 1 |
| FMS682 | het | FMS684 | wt | FMS683 | het | 0 | 1 |
| FMS38 | wt | FMS36 | wt | FMS37 | het | 1 | 0 |
| FMS137 | wt | FMS141 | het | FMS140 | wt | 1 | 0 |
| FMS321 | wt | FMS322 | wt | FMS323 | het | 1 | 0 |
| FMS367 | wt | FMS369 | het | FMS370 | wt | 1 | 0 |
| FMS495 | wt | FMS497 | het | FMS496 | wt | 1 | 0 |
| FMS659 | wt | FMS661 | het | FMS660 | wt | 1 | 0 |
| FMS679 | wt | FMS681 | het | FMS680 | wt | 1 | 0 |
| **Total** | | | | | | **7** | **17** |

^1^ het= heterozygous, wt= wild type; ut= untransmitted, t= transmitted; P= 0.032.
